# Oral Microbiota Shift after 12-Week Supplementation with *Lactobacillus reuteri* DSM 17938 and PTA 5289; A Randomized Control Trial

**DOI:** 10.1371/journal.pone.0125812

**Published:** 2015-05-06

**Authors:** Nelly Romani Vestman, Tsute Chen, Pernilla Lif Holgerson, Carina Öhman, Ingegerd Johansson

**Affiliations:** 1 Department of Odontology/section of Cariology, Umeå University, Umeå, Sweden; 2 Department of Microbiology, The Forsyth Institute, Cambridge, United States of America; 3 Department of Odontology/section of Pedodontics, Umeå University, Umeå, Sweden; GI Lab, UNITED STATES

## Abstract

**Background:**

*Lactobacillus* spp. potentially contribute to health by modulating bacterial biofilm formation, but their effects on the overall oral microbiota remain unclear.

**Methods and Findings:**

Oral microbiota was characterized via 454-pyrosequencing of the 16S rDNA hypervariable region V3-V4 after 12 weeks of daily *Lactobacillus reuteri* DSM 17938 and PTA 5289 consumption. Forty-four adults were assigned to a test group (n = 22) that received lactobacilli lozenges (10^8^ CFU of each strain/lozenge) or a control group that received placebo (n = 22). Presence of *L*. *reuteri* was confirmed by cultivation and species specific PCR. Tooth biofilm samples from 16 adults before, during, and after exposure were analyzed by pyrosequencing. A total of 1,310,292 sequences were quality filtered. After removing single reads, 257 species or phylotypes were identified at 98.5% identity in the Human Oral Microbiome Database. *Firmicutes*, *Bacteroidetes*, *Fusobacteria*, *Proteobacteria*, and *Actinobacteria *were the most abundant phyla. *Streptococcus* was the most common genus and the *S*. *oralis/S*. *mitis/S*. *mitis bv2/S*. *infantis* group comprised the dominant species. The number of observed species was unaffected by *L*. *reuteri* exposure. However, subjects who had consumed *L*. *reuteri* were clustered in a principal coordinates analysis relative to scattering at baseline, and multivariate modeling of pyrosequencing microbiota, and culture and PCR detected *L*. *reuteri* separated baseline from 12-week samples in test subjects. *L*. *reuteri* intake correlated with increased *S*. *oralis/S*. *mitis/S*. *mitis bv2/S*. *infantis* group and *Campylobacter concisus*, *Granulicatella adiacens*, *Bergeyella sp*. HOT322, *Neisseria subflava*, *and SR1 [G-1] sp*. HOT874 detection and reduced *S*. *mutans*, *S*. *anginosus*, *N*. *mucosa*, *Fusobacterium periodicum*, *F*. *nucleatum ss vincentii*, and *Prevotella maculosa *detection. This effect had disappeared 1 month after exposure was terminated.

**Conclusions:**

*L*. *reuteri* consumption did not affect species richness but induced a shift in the oral microbiota composition. The biological relevance of this remains to be elucidated.

**Trial Registration:**

ClinicalTrials.gov NCT02311218

## Introduction

The oral cavity provides distinct niches for bacterial communities, including saliva, gingival fluid, and food-flushed keratinized/non-keratinized epithelial or mineralized tooth surfaces [1]. These communities harbor both disease- and health-associated species. In the oral cavity, approximately 700 prevalent taxa have been identified in the mouth [www.homd.org; 2]; however, the core microbiome is dominated by *Firmicutes*, which includes both streptococci and lactobacilli [2]. Lactobacilli, which are commonly used in health promoting products (probiotics) [3] and industrial and artisanal food and dairy fermentation [4], have been reported to influence immune responses, nutrition, and overall wellbeing [5]. Moreover, Lactobacilli are characterized by lactic acid production and a tolerance for low pH; although these features may be advantageous for modulating the microbial ecology in some body sites, they confer cariogenic traits on oral lactobacilli [6]. However, recent studies have suggested the following possible beneficial effects of oral lactobacilli: (*i*) *in vitro* growth inhibition of laboratory strains and clinical isolates of cariogenic *Streptococcus mutans* and *S*. *sobrinus* [7], (*ii*) association between some lactobacilli species and healthy teeth [8], and (*iii*) *in vitro* growth and attachment inhibition of various opportunistic oral bacteria by *Lactobacillus gasseri* isolated from the mouths of 3–4-month-old breast-fed infants [9,10].

*The bacterium* L. reuteri is indigenous in humans [11] and can be isolated from the gastrointestinal tract, vagina, and breast milk [12,13]. *L*. *reuteri* 55730 (isolated from breast milk) as well as its daughter strain DMS 17938 (differs from 55730 by the removal of tetracycline and lincosamide resistance gene-bearing plasmids) [14] are well-documented probiotic strains. Thus, these strains have been reported to improve gut function in pre-term infants [15], ease symptoms of infantile colic [16], and reduce constipation or pain in children with abdominal dysfunction [17]. Further, genome-wide analysis and transcriptome comparisons among lactobacilli support probiotic features for *L*. *reuteri* 55730 [18]. PTA 5289 (human oral cavity isolate) is another well-documented probiotic *L*. *reuteri* strain [19] that is particularly characterized by its immunosuppressive properties [20], such as the suppression of tumor necrosis factor (TNF) production by lipopolysaccharide (LPS)-activated monocytoid cells [20]. Furthermore, *L*. *reuteri* PTA 5289 can adhere to human intestinal cells [21] and bind to saliva-coated hydroxyapatite *in vitro* (a model for tooth enamel) [22]. McNulty et al. [23] have shown that probiotic bacterial species from fermented milk alter the bacteria metatranscriptome but not species profile in twins and gnotobiotic mice.

Several studies have reported reduced mutans streptococci in saliva after short-term *L*. *reuteri* consumption [24,25], but this effect has not been uniformly observed [26,27]. However, it remains unclear if *L*. *reuteri* consumption affects the overall oral microbiota composition. Considering the complexity of oral microbiota and the unavailability of support for reducing oral disease outcomes, recommendations regarding *L*. *reuteri* consumption for oral health purposes remain unsubstantiated.

High-throughput sequencing of bacterial 16S rRNA genes, such as the 454-amplicon pyrosequencing technique [28] combined with taxonomic assignment in the curated 16S rRNA Human Oral Microbiome Database (HOMD, www.homd.org) [29], facilitate bacterial mapping at the species level in complex oral ecosystems [30]. This level of taxonomic resolution is desired for oral microbiota as some species within a genus, such as those in *Streptococcus*, are known to be associated with health, whereas others have been linked to disease development [31]. The current study aimed to evaluate the effects of a 12-week *L*. *reuteri* (DSM 17938 and PTA 5289) supplementation regimen on the oral microbiota composition using 454 pyrosequencing and the HOMD combined with *L*. *reuteri* specific culturing and PCR detection according to the hypothesis that probiotic *L*. *reuteri* induces a shift in oral microbiota.

## Materials and Methods

The protocol for this trial and supporting CONSORT checklist are available as supporting information; see S1 CONSORT Checklist and S1 Protocol.

### Ethics statements

The study was approved on Jan 11, 2012 by the Regional Ethical Review Board in Umeå, Sweden (Dnr 2011-380-31M) and was conducted according to the principles expressed in the Declaration of Helsinki. Written informed consent was obtained from all participants.

### Subjects and study design

Healthy adult volunteers, aged 20–66 years, among students and employees at the Faculty of Medicine, Umeå University, Sweden were recruited to a double-blind, randomized controlled trial (RCT) through advertisements. Subjects were recruited in January 2013, the intervention period started in March 2013, and follow-ups were done in June and August 2013. The study has been registered at https://clinicaltrials.gov/ (NCT02311218) after the study was completed. The reason for this is that it was requested by the journal, but it is not compulsory for approval by the Swedish Ethical Review Boards. The authors confirm that all ongoing and related trials for this intervention are registered. Inclusion criteria were a self-reported healthy status and no use of antibiotics or probiotic products during 3 months prior to the study. Based on previous studies regarding the persistence of probiotic strains [32,33], the recruitment goal was at least 15 people per study group.

**Fig 1:**
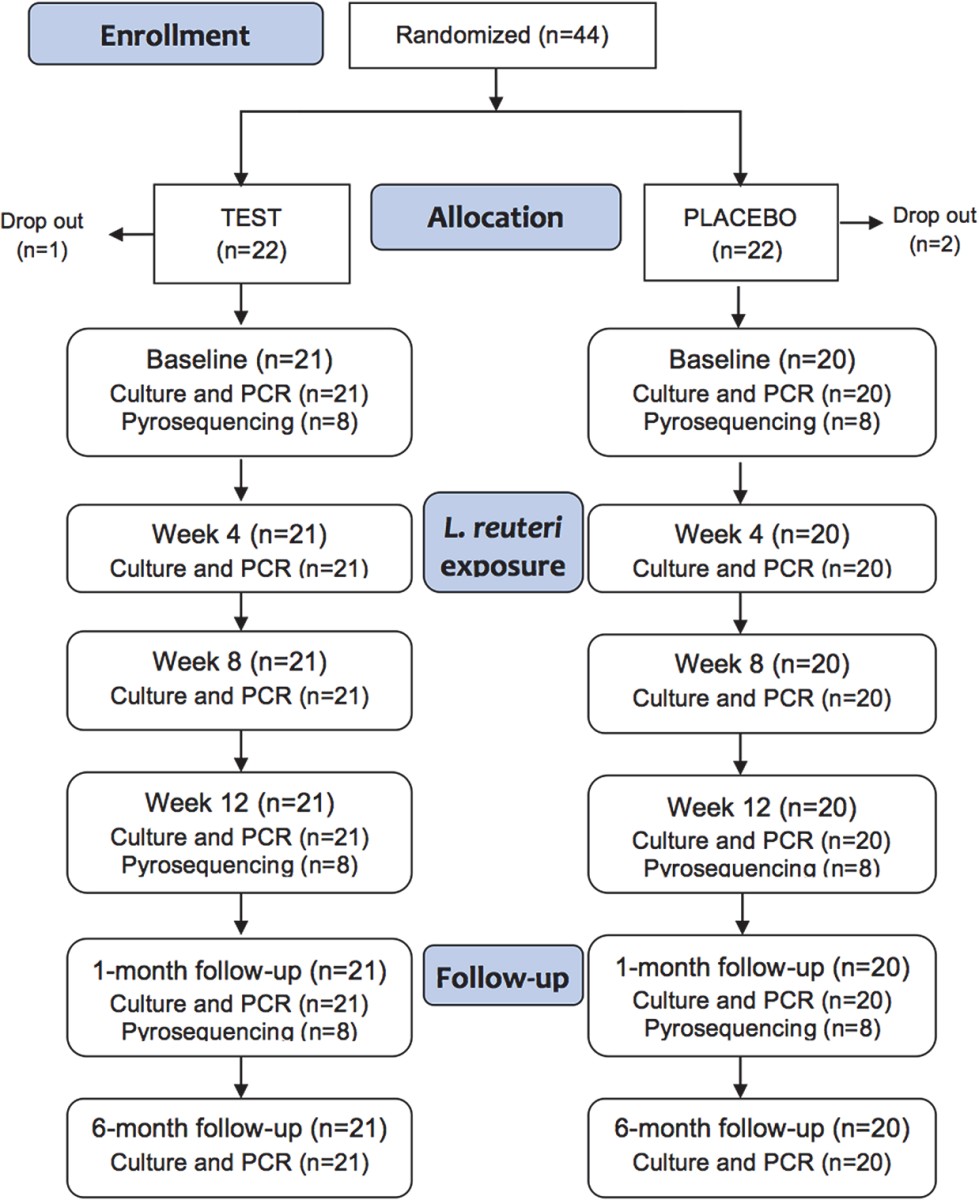
Flow chart diagram. Number of study participants and samples analyzed in the test and placebo groups at each sampling occasion.

Forty-four volunteers, none of whom used tobacco products or were co-habitants, were recruited and randomly allocated to either a test (n = 22) or placebo group (n = 22; Fig 1). SPSS (version 22.0, IBM Corporation, Armonk, NY, USA) was used for randomization and group allocation. This was performed by a person who did not participate in sample collection, breaking the codes or data input. Correct implementation of the group allocation was secured by sequentially numbering of differentially colored medical trays used for lozenge distribution. All participants had at least 28 teeth and none had any clinically detectable sign of dental caries or periodontal disease. All participants brushed their teeth twice a day using commercially available fluoride containing toothpaste. Participants were asked to allow 2 lozenges per day to slowly melt in the mouth and to circulate the dissolved tablet contents around their mouths. One lozenge was taken in the morning and 1 in the evening for 12 weeks. The participants were instructed to take the lozenges after the morning meal and tooth brushing and in the early evening well before tooth brushing at bed time. They were instructed to maintain their regular oral hygiene regimen except for the 48 hour preceding the sampling occasions. The test lozenges contained *L*. *reuteri* (DSM 17938 and PTA 5289; 10^8^ CFU per strain; BioGaia AB, Stockholm, Sweden), isomalt, hydrogenated palm oil, peppermint and menthol flavoring, peppermint oil, and sucralose (http://www.biogaia.com/product/biogaia-prodentis-oral-lozenges). The placebo lozenges were identical to the test lozenges in appearance, taste, and composition except the lactobacilli. The content of the lozenges was blinded to the participants, to the personal who exchanged the medical trays and sampled, and the personal who handled and analyzed the samples. Compliance was monitored as the percentage of lozenges consumed of the total assigned number. The remaining lozenges were counted when the containers were returned for monthly refills to assess this factor. Compliance was considered acceptable if ≤15% of the lozenges remained. Participants were asked to abstain from oral hygiene for 48 h and to not consume any food for at least 4 h before sampling. Participants were also instructed to not eat probiotic products throughout the study period.

### Saliva and biofilm sampling

Saliva and tooth biofilm samples were obtained immediately before (baseline) and after 4, 8, and 12 weeks of *L*. *reuteri* supplementation (Fig 1). Follow-up samples were collected 1 and 6 months after supplementation was terminated. Furthermore, whole stimulated saliva (~5 mL) was generated by chewing 1 g of paraffin and collected into ice-chilled sterile test tubes. One milliliter of saliva was used for cultural analysis, and the remaining saliva was centrifuged at 3,500 × *g* for 10 min at 4°C. The pellets were stored at −80°C until DNA extraction for strain-specific PCR reactions. For the pyrosequencing analysis, pooled supragingival plaque was collected with sterilized toothpicks and transferred to Eppendorf tubes (Sarstedt, Nümbrecht, Germany) containing 200 μL of TE-buffer (10 mM Tris, 1 mM EDTA, pH 7.6). The samples were stored at −80°C until DNA extraction.

### Identification of lactobacilli by culture and PCR

Aliquots of saliva were plated onto Rogosa agar (Merck, Darmstadt, Germany) to obtain *Lactobacillus* counts and on selective agar for tentative identification of the *L*. *reuteri* (DSM 17938 and PTA 5289) strains [34]. All plates were anaerobically incubated at 37°C for 48–72 h, except *L*. *reuteri* PTA 5289, which was anaerobically incubated at 40°C for 72 h.

DNA was extracted from saliva pellets as described [35]. *L*. *reuteri* DSM 17938 and PTA 5289-specific PCR were identified using KAPA2G Robust HotStart PCR Ready Mix (2×) (Kapa Biosystems, Boston, MA, USA) and strain-specific primers [34]. Briefly, 2 μL of DNA extract was added to a total reaction volume of 25 μL (containing 12.5 μL of master mix and each primer pair at a concentration of 0.5 μM). PCR conditions were 95°C for 3 min; 40 cycles of 95°C for 15 s, 60°C for 15 s, and 72°C for 30 s; and 72°C for 5 min. PCR products were then verified by electrophoresis on 2% agarose gels allowed to run for 80 min at 120 V in 0.5× TBE (Tris/Borate/EDTA) buffer, pH 8.3, followed by ethidium bromide (0.2 μg/μL) staining.

### Pyrosequencing analysis

For the 454 pyrosequencing analysis, 16 subjects were randomly selected among the test (n = 8) and control (n = 8) subjects (Fig 1).

DNA was extracted from the baseline, 12-week exposure, and 1-month follow-up tooth biofilm samples as previously described [35]. The V3-V4 hypervariable region of the 16S rRNA gene was amplified via PCR using the forward primer 347F and reverse primer 803R [36]. For sample identification, fusion primers were created from these primers and unique barcode sequences according to the Roche guidelines for experimental amplicon design (www.454.com). DNA was amplified under the following running conditions: initial denaturation at 94°C for 3 min; 30 cycles of 94°C for 15 s, 58°C for 15 s, and 72°C for 30 s; and a final extension at 72°C for 8 min.

Amplicon processing and 454 sequencing were conducted at the Lund University Sequencing Facility (Faculty of Science, Lund, Sweden). After amplicon cleaning to remove short fragments (Agencourt AMPure XP; Beckman Coulter, Brea, CA, USA) and inspection (DNA 1000 kit on a 2100 Bioanalyzer; Agilent Technologies, Palo Alto, CA, USA), amplicons were quantified (Quant-iT ds DNA assay kit; Invitrogen, Carlsbad, CA, USA and Quantifluor fluorometer; Promega, Madison, WI, USA) and diluted to obtain a total of 10^7^ copies/μL. Titration and library production (target: 10%–15% enrichment) were performed using emulsion PCR and the Lib-A kit (Roche Diagnostics, Branford, CT, USA). DNA-positive beads were enriched, counted (Innovatis CASY particle counter; Roche Innovatis, Bielefeld, Germany), processed (XLR70 sequencing kit; Roche Diagnostics), and loaded onto a picotiter plate for pyrosequencing on a 454 Life Sciences Genome Sequencer FLX+ machine (Roche Applied Sciences; Penzberg, Germany).

### Data processing

Subject characteristic, compliance, and culture data were processed using SPSS. Descriptive statistics, such as means [95% confidence intervals (CI)] and proportion distributions were calculated. Differences between groups were tested with parametric or non-parametric tests depending on the data measurement and distribution levels. P < 0.05 was considered statistically significant.

Sequences with a minimum length of 300 base pairs after primer sequence removal, correct barcode sequences, a maximum of 1 incorrect base pair in the primer sequences, and compliance with the default quality criteria for homopolymers and quality scores in the Quantitative Insights into Microbial Ecology (QIIME) [37] software package (version 1.8.0) were retained for analysis. Any sequence beyond the reversed primer were removed. Sequences beginning with the reverse primer were reverse complemented. Sequences were then clustered into operational taxonomic units (OTUs) at a sequence similarity of 97% in the 16S rRNA chimera-checked Greengene database (dated May 2013) [38] using USEARCH [39]. OTUs with a single sequence were removed. One representative sequence per remaining OTU was taxonomically assigned as a named or unnamed cultivable species or uncultivable phylotype at ≥98.5% identity using the HOMD 16S rRNA RefSeq, version 12.0 (http://www.homd.org) [29].

Rarefaction curves were calculated to compare microbial richness [40]. Principal coordinate analysis (PCoA) was applied to evaluate the phylogenetic beta diversity [41] of the bacterial profiles at different time points. Multivariate partial least-square analysis (PLS) modeling (SIMCA P+, version 12.0; Umetrics AB, Umeå, Sweden) was performed to search if microbiota structures were related to *L*. *reuteri* consumption (y-variables) [42,43]. Tested models included those with pyrosequencing data only and those to which lactobacilli and streptococci culture and PCR data had been added. Variables were autoscaled to unit variance and cross-validated Y predictions were calculated. Subject clustering was displayed in score loading plots, and the importance of each x-variable was displayed in loading plots. Variables, where the 95% CI of the PLS correlation coefficient did not inlude zero were considered statistically significant [43]. The Q^2^ value yielded the capacities of the x-variables to predict the outcome (test or placebo group allocation). Univariate analyses of single taxa were not applied because of the combination of small groups and a high number of repeated tests.

## Results

### Study group and lactobacilli retrieval by culture

Saliva and plaque samples were collected on 6 different occasions from 41 subjects in either a test group in which subjects ingested lozenges containing *L*. *reuteri* or a control group in which subjects ingested placebo lozenges (Fig 1). Three participants (1 from the test and 2 from the placebo group) dropped out before baseline sampling for personal reasons. None reported any unintended effect from taking the lozenges. No significant differences were found in age, sex, or compliance with the study protocol between the 2 groups (Table 1).

**Table 1:** Characteristics of participants by *L*. *reuteri* consumption.

Variable	Placebo (n = 20)		Test (n = 21)		*P*-value	
**Gender (% men)** ^**1**^	53.3		46.7		0.453	
**Age in years [mean (95% CI)]** ^**2**^	27.8 (23.8–31.8)		28.5 (23.4–33.7)		0.819	
**Compliance [mean (95% CI)]** ^**2**^ ^,^ ^**3**^	90.2 (86.3–94.1)		97.8 (89.3–96.2)		0.100	
**Lactobacilli positive by culture [n (%)]** ^**1**^						
baseline	6 (30.0)		6 (28.6)		0.595	
week 4	6 (30.0)		17 (81.0)		0.001	
week 8	8 (40.0)		14 (66.7)		0.081	
week 12	6 (30.0)		18 (85.7)		<0.001	
1-month follow-up	5 (25.0)		11 (52.4)		0.069	
**Lactobacilli counts [log CFU/mL saliva, mean (95%CI)]** ^**4**^						
baseline	0.36 (0.07–0.66)		0.42 (0.05–0.80)		0.796	
week 4	0.84 (0.19–1.49)		2.54 (1.88–3.21)		<0.001	
week 8	1.04 (0.39–1.68)		1.96 (1.26–2.66)		0.049	
week 12	0.72 (0.17–1.28)		2.61 (1.94–3.28)		<0.001	
1-month follow-up	0.68 (0.07–1.28)		1.61 (0.86–2.36		0.051	
***L*. *reuteri* DSM 17938 [n (%) positive]** ^**1**^	**Culture**	**PCR**	**Culture**	**PCR**	**Culture**	**PCR**
Baseline	0	0	0	0	-	-
week 4	0	0	10 (47.6)	13 (61.9)	<0.001	<0.001
week 8	0	0	14 (66.7)	15 (71.4)	<0.001	<0.001
week 12	0	0	13 (61.9)	15 (71.4)	<0.001	<0.001
1-month follow-up	0	0	4 (19.0)	6 (28.6)	0.059	0.012
6-month follow-up	0	0	0	0	-	-
***L*. *reuteri* PTA 5289 [n (%) positive]** ^**1**^	**Culture**	**PCR**	**Culture**	**PCR**	**Culture**	**PCR**
baseline	0	0	0	0	-	-
week 4	0	1 (5.0)	5 (23.8)	15 (71.4)	0.027	<0.001
week 8	0	0	4 (19.0)	13 (61.9)	0.059	<0.001
week 12	0	0	9 (42.9)	14 (66.7)	0.001	<0.001
1-month follow-up	0	0	2 (9.5)	4 (19.0)	0.059	0.256
6-month follow-up	0	0	0	0	-	-

^1^ Differences between group numbers tested with Chi^2^ test

^2^ Differences between group means tested with Student’s *t*-test

^3^ % compliance = (lozenges consumed/ lozenges expected to be consumed)*100

^4^ Difference between the test and placebo group were analyzed by non-parametric statistics (The Mann–Whitney U-test). Numbers are expressed as log_10_ values

CFU, colony-forming unit; CI, confidence interval

At baseline, lactobacilli were cultivated from saliva in 28.6% and 30.0% (p = 0.595) of participants in the test and placebo groups, respectively (Table 1). During exposure, the test group had a larger proportion of participants with cultivable lactobacilli than did the placebo group (p < 0.001). Moreover, mean numbers of log CFU/mL in saliva significantly differed between the test and placebo groups during the exposure period. However, 1-month follow-up values in the test group for both the proportion with cultivable lactobacilli and the mean numbers of log CFU/mL in saliva approached the baseline values (Table 1).

*L*. *reuteri* DSM 17938 was not detected in the placebo group at any time point, whereas *L*. *reuteri* PTA 5289 (or a highly similar strain) was detected in 1 participant at week 4 (Table 1). Similarly, no participants in the test group carried any of the *L*. *reuteri* test strains at baseline or at the 6-month follow-up. During exposure, mean PCR-detected prevalence rates of the *L*. *reuteri* strains DSM 17938 and PTA 5289 were 68.2% and 66.7%, respectively. At the 1-month follow-up, DSM 17938 and PTA 5289 remained detectable in 28.6% and 19.0% of the test participants, respectively (Table 1). *L*. *reuteri* was consistently more frequently detected by PCR than by culture (Table 1).

### Sequencing output

A total of 1,310,292 reads were obtained for the 48 tooth biofilm samples, *i*.*e*. 3 samples for 8 subjects in two groups. The original read sequences and the OUT table is available at ftp://www.homd.org/publication_data/20150122. Sequencing failed in 1 test group sample and that subject was excluded from all analyses. For the remaining 15 participants, quality control and denoising reduced the numbers to 1,148,923 sequences, out of which, 367,596, 422,639, and 358,688 corresponded to the baseline, 12-week exposure, and 1-month follow-up samples, respectively. The number of reads per sample ranged from 10,190 to 54,500 (mean, 26,690; median, 22,699), and the average read length was 415 base pairs. A total of 257 named or unnamed species or uncultivable phylotypes with at least 2 sequences per cluster were taxonomically assigned at 98.5% identity in the HOMD. These represented 9 phyla and 66 genera (S1 Table).

### Dental biofilm composition at baseline

UCLUST clustered 367,596 sequences from the baseline samples into 1,221 OTUs with ≥2 reads per cluster against the Greengene database at ≥97% sequence similarity. These matched with 1,011 sequences in the HOMD at ≥98.5% identity, representing 223 unique named or unnamed species or uncultivable phylotypes in the HOMD. Among these, 119 were named species, 55 were unnamed species, and 49 were uncultivable phylotypes. Under these restrictions, an average of 101 species or phylotypes were identified per baseline sample.

**Fig 2:**
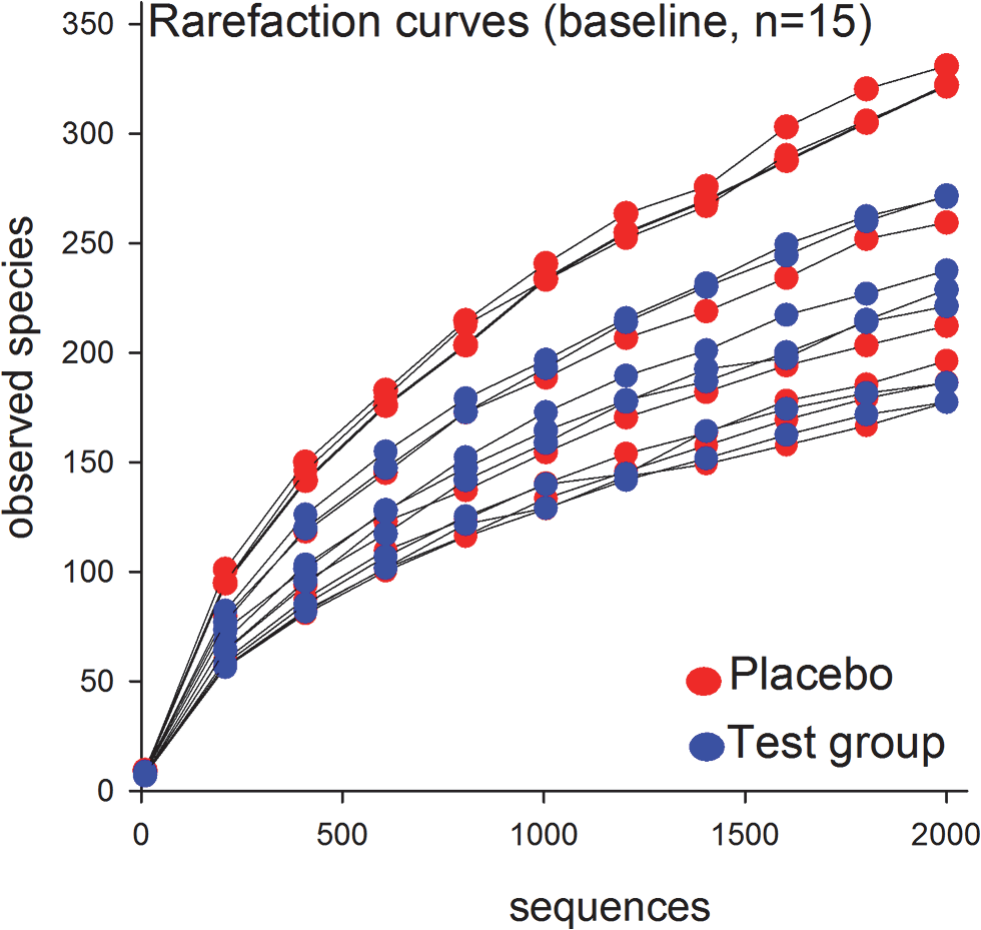
Rarefaction curves of baseline operational taxonomic unit prevalence rates according to the number of reads. Comparisons include all 15 subjects at baseline. Red dots indicate subjects in the placebo group; blue dots indicate subjects in the test group.

Rarefaction curves showed a 2-fold difference in sequence richness among the 15 baseline samples (Fig 2). Although this variation was reflected in individually differing proportions at the phylum and genus levels, the phyla and genera prevalence rankings in all subjects basically followed the pattern described below.

**Fig 3:**
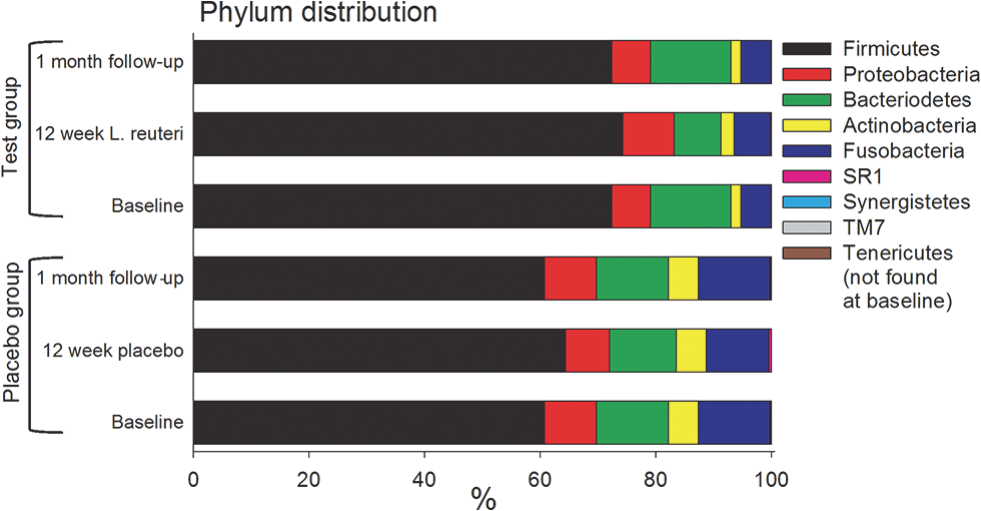
Relative abundances of 9 identified bacterial phyla in 45 plaque samples. Sequences were matched to Human Oral Microbiome Database using Quantitative Insights into Microbial Ecology (QIIME) for phylum-level taxonomic identification. Data are presented as stacked bars and phyla are ordered by decreasing abundance and stratified by study group and sample occasion (*i*.*e*., baseline, 12-week exposure, and 1-month follow-up).

The 223 HOMD identified species/phylotypes in the baseline samples represented 8 phyla and 55 genera (Fig 3 and S2 Table). *Firmicutes* (65.1%) was the most prevalent phylum, followed by *Bacteroidetes* (13.0%), *Fusobacteria* (9.7%), *Proteobacteria* (8.1%), and *Actinobacteria* (3.9%*)*. *SR1*, *TM7*, and *Synergistetes* each represented <1% of the sample sequences. At the genus level, *Streptococcus* was most common (44.9%), followed by *Fusobacterium*, *Veillonella*, *Haemophilus*, *Selemonas*, *Capnocytophaga*, *Abiotrophia*, *Leptotrichia*, *Prevotella*, *Porphyromonas*, *Actinomyces*, *Gemella*, *and Granulicatella* (prevalence range: 2.1%–5.6%). The remaining 42 genera were detected at <1% abundance. At the species level, the *S*. *oralis/S*. *mitis/S*. *mitis bv2/S*. *infantis* group was most common (26.3%), followed by *S*. *sanguinis* (7.5%), *Haemophilus parainfluenzae* (4.9%), *S*. *oligofermentans* (4.2%), *Abiotrophia defectiva* (4.2%), *Veillonella dispar* (3.6%), and *Fusobacterium nucleatum ss*. *polymorphum* (3.1%). Furthermore, *S*. *gordonii*, *Porphyromonas sp*. HOT279, *Capnocytophaga sputigena*, *S*. *peroris*, *Gemella morbillorum*, *Granulicatella adiacens*, *Veillonella parvula*, and *Selemonas sp*. HOT137 each represented between 1% and <3% of the sequences; the remaining 208 species each represented <1% of all sequences.

### Stability in placebo-treated subjects

**Fig 4:**
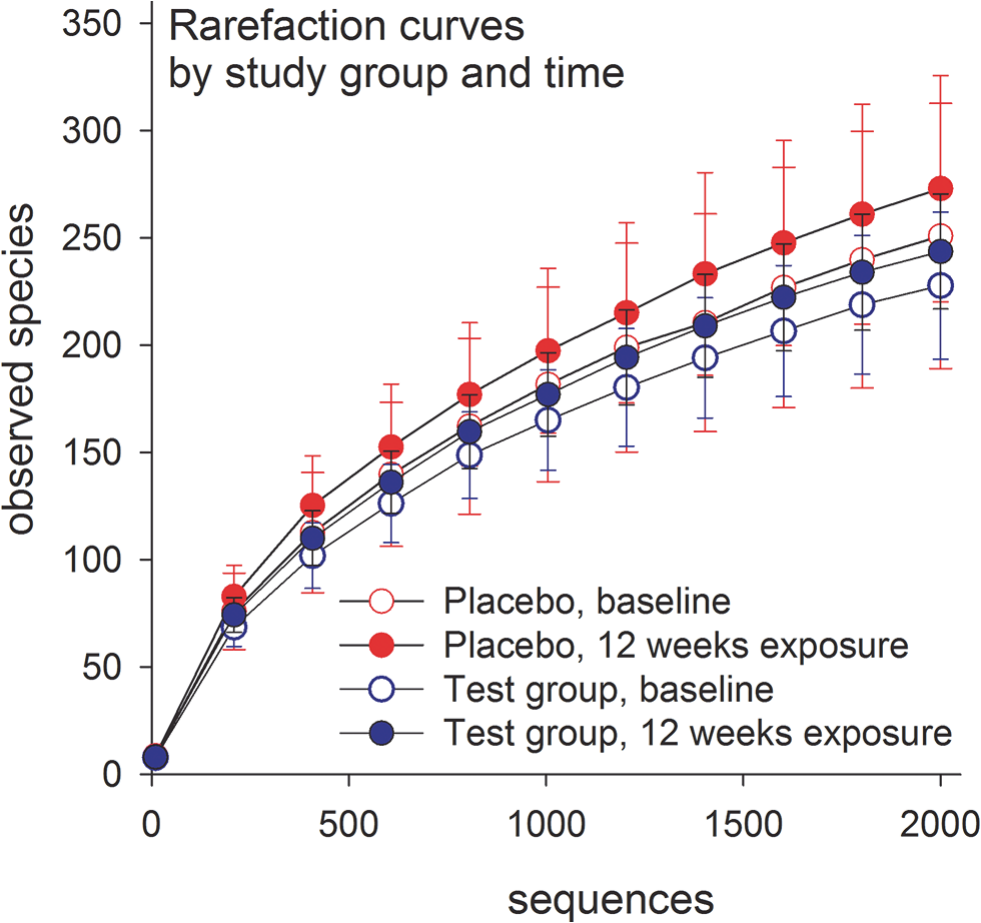
Rarefaction curves of operational taxonomic unit prevalence rates according to the number of reads in the test and placebo groups. Data are presented as means with standard errors. Comparisons include 8 subjects in the placebo-treated group by sampling occasion (red symbols) and 7 subjects in the test group by sampling occasion (blue symbols). Differences within or between the groups were not statistically significant.

**Fig 5:**
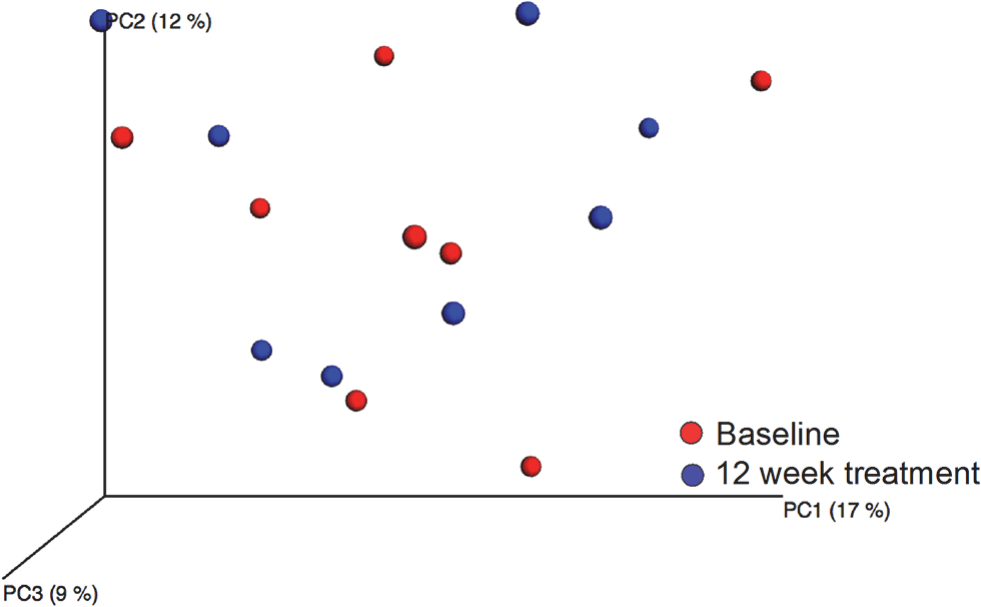
PCoA clustering analysis of baseline and 12-week samples in the placebo group. Red dots indicate baseline samples and blue dots 12 week samples.

Microbiota compositions of the dental biofilm samples from the placebo group were stable over the 12-week treatment period, as determined by the lack of difference in the number of observed species between the baseline and 12-week treatment samples (Fig 4), the lack of clustering in PCoA modeling of beta-diversity in the baseline and 12-week samples (Fig 5), and the lack of a significant model obtained by multivariate PLS modeling in which sampling occasion (baseline versus 12-week treatment samples) was the dependent variable and phyla, genera, and species assignments were the independent variables (data not shown).

### Effect of 12-week *L*. *reuteri* treatment

**Fig 6:**
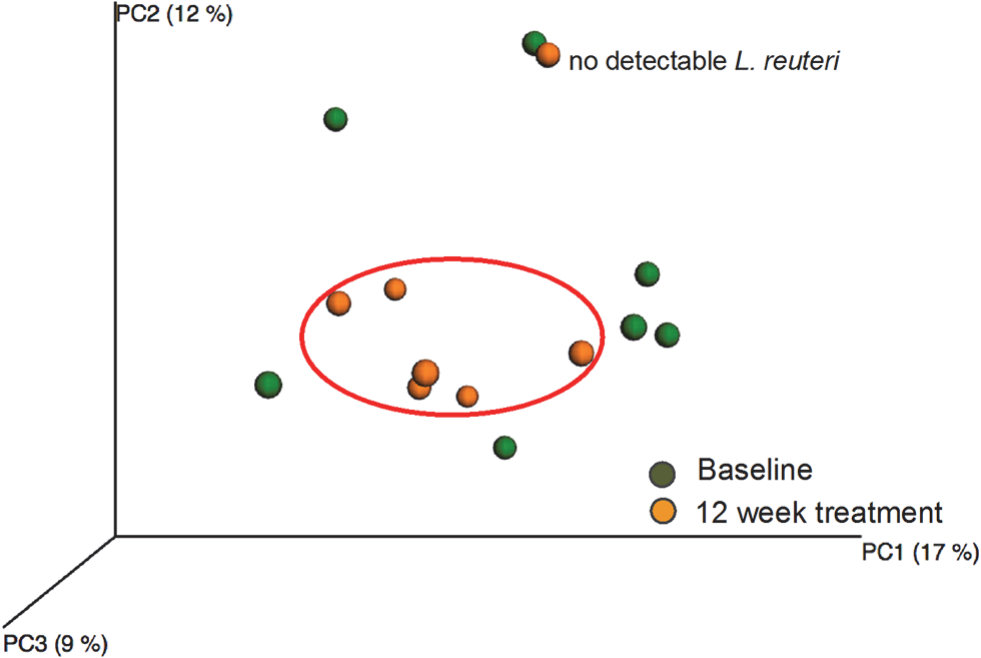
PCoA clustering analysis of baseline and 12-week samples in the test group. Green dots indicate baseline samples and yellow dots 12 week samples.

**Fig 7:**
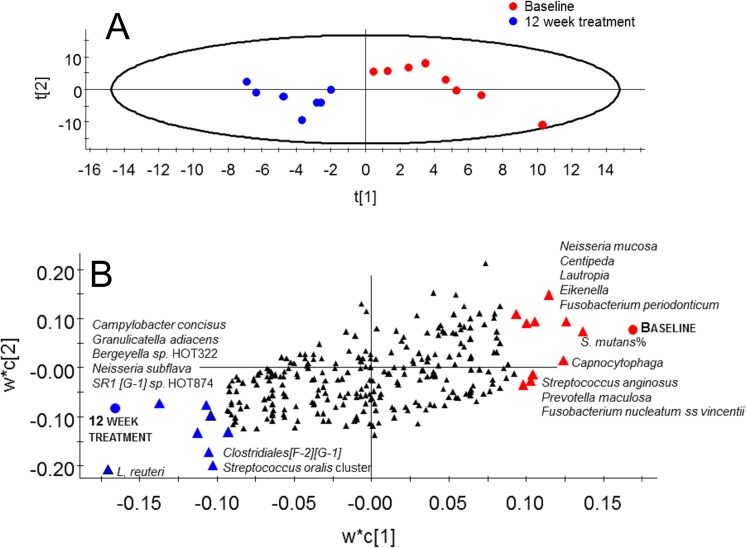
Partial least-squares analysis (PLS) of microbiota associated with 12 weeks consumption of *L*. *reuteri*. (A) Scatter-loading plot illustrating clustering of baseline versus 12 week treatment samples from the test group; The scores t1 and t2 are the new PCA created variables summarizing the x-variables. The red dots indicate placebo samples and blue dots test samples. The oval circle illustrates the tolerance ellipse based on Hotelling´s of T2, any observation located outside of the elipse would be an outlier. (B) Loading plot illustrating taxa associated with baseline versus 12 week treatment in the test group. The PLS model employed test and placebo group allocation as y and pyrosequencing taxa, *L*. *reuteri* and *S*. *mutans* by cultivation and PCR as the x-block. Red triangles indicate taxa associated with the baseline microbiota in the test group and blue triangles those associated with the microbiota after 12 week treatment in the same group. Black symbols indicate variables that were non-influential in the projection.

Similar to the placebo group, the number of observed species did not differ between baseline and 12-week treatment samples in the test group (Fig 4). However, PCoA modeling of the phylogenetic divergence among the UCLUST-identified OTUs clustered all 12-week treated samples together except for 1 sample (Fig 6). Notably, that sample was from the only participant in the test group (used for pyrosequencing) without detectable *L*. *reuteri* DMS 17938 or PTA 5289 at any sampling occasion. In contrast, the baseline samples did not cluster. PLS modeling using species/phylotypes and culture/PCR data yielded a model with 1 significant component and a predictive power (Q^2^) of 43% for having ingested *L*. *reuteri* bacteria for 12 weeks relative to the baseline. This model fully separated the 12-week treatment samples of the test subjects from their baseline samples (Fig 7A). Baseline samples were associated with a higher proportion of *S*. *mutans* among the total streptococci count along with higher detection frequencies of the species *N*. *mucosa*, *F*. *periodicum*, *F*. *nucleatum ss vincentii*, *S*. *anginosus*, and *Prevotella maculosa* (Fig 7B). Presence of the test bacteria (*L*. *reuteri*) and greater detection frequency of the species *Campylobacter concisus*, *G*. *adiacens*, *Bergeyella sp*. HOT322, *N*. *subflava*, *SR1 [G-1] sp*. HOT874, and the *S*. *oralis/S*. *mitis/S*. *mitis bv2/S*. *infantis* group were associated with consumption of the *L*. *reuteri*-containing lozenges for 12 weeks (Fig 7B). Restricting the model to include only pyrosequencing obtained species/phylotypes did not alter the overall results.

Prevalence rates at the phylum and genus level as well as the species/phylotype level in the test and placebo groups are presented in S1–S2 Tables, respectively.

### No persistent treatment effect at 1-month follow-up

**Fig 8:**
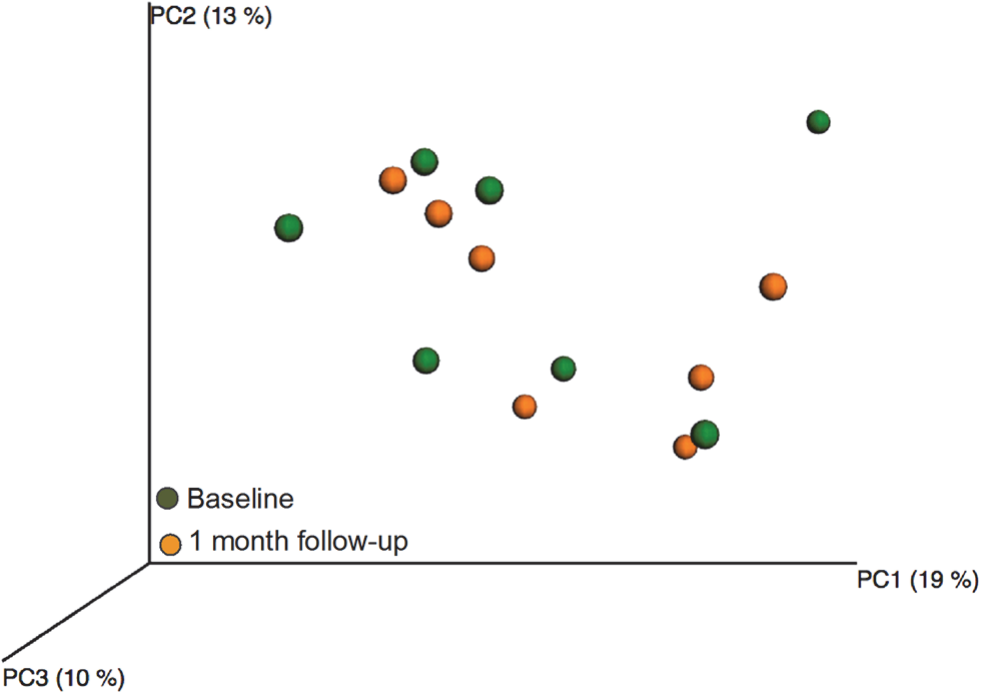
PCoA clustering analysis of 12-week and 1 month follow-up samples in the test group. Yellow dots indicate baseline samples and green dots 1 month follow-up samples.

Rarefaction curves showed that in the test group, species richness in the 1-month follow-up samples did not differ from that in the baseline or 12-week treatment samples (data not shown). The PCoA projection of beta diversity in the baseline samples versus the 1-month follow-up samples showed no sample clustering, such as that observed in the 12-week treatment samples (Fig 8). Further, no significant components were found by PLS using species/phylotypes with or without lactobacilli and mutans streptococci from culture and PCR as the independent block and sampling occasion as dependent variables.

## Discussion

The present study evaluated if consuming the probiotic *L*. *reuteri* strains DMS 17938 and PTA 5289 for 12 weeks would modify the microbiota composition and species richness in tooth biofilms. The main findings were as follows: (*i*) the composition shifted but species richness remained unaffected, (*ii*) the shift normalized within 1 month after terminating exposure, and (*iii*) the test strains could not be detected in approximately 30% of the participants despite receiving daily booster doses.

The ecology of the gastrointestinal microbiota, including that of the mouth, is virtually stable once the period of variation in early childhood has passed [44]. Previous multiplex characterizations of the microbiota following exposure to probiotic bacteria or dietary regimens have essentially addressed the impact on the gut microbiome [45, 46]. Studies that have addressed effects on oral bacterial communities have mainly targeted single bacterial species, such as the caries-associated mutans streptococci (*S*. *mutans* and *S*. *sobrinus)* [24–27]. *L*. *reuteri* DSM 17938 and PTA 5289 ingestion have been previously associated with reduced oral levels of mutans streptococci [24,25]. However, several studies found no evidence of reduced disease development despite reductions in mutans streptococci levels [47,48], the mechanisms of which include growth inhibition and competition for host ligands, such as gp340 [49–51]. In the present study, the proportion of *S*. *mutans* among total streptococci was lower in the test group after a 12-week period during which *L*. *reuteri* strains DSM 17938 and PTA 5289 were ingested. This finding partly reflected a reduction in mutans streptococci in some subjects (culture data; Johansson *et al*, manuscript in progress) and partly reflected the increased numbers of other streptococci (*e*.*g*., the *S*. *oralis/S*. *mitis/S*. *mitis bv2/S*. *infantis* group). However, this result should be interpreted with caution as mutans streptococcal colonization was not an inclusion criterion in the present study, in contrast to the referred studies [24,25] and few subjects were found to be colonized with *S*. *mutans* (none were colonized with *S*. *sobrinus*). A potentially beneficial effect of *L*. *reuteri* exposure was the reduced prevalence of *F*. *nucleatum ss vincentii* and *F*. *periodicum*, which may be linked to reduced biofilm formation and gingivitis development during the ingestion of probiotic bacteria [52]. Fusobacteria are considered a key link between early and late colonizers in oral biofilm formation due to the ability to coaggregate with a large number of bacterial species, including probiotic bacteria [1,61].

Two previous studies evaluated oral microbiota after the short-term use (3 or 4 weeks) of probiotic bacteria [52,53]. One study incorporated a whole-genome DNA–DNA hybridization technique [53], whereas the other used a specially designed 16S rRNA-based microarray (HOMIM) [52]. However, neither study found an ecological shift. The present study, which exposed subjects to *L*. *reuteri* for a longer period (12 weeks) and mapped the microbiota by sequencing an approximately 400-base pair section of the 16S rRNA gene, revealed a transient reduction in diversity in the tooth biofilm samples, although this shift normalized within 1 month after intake was discontinued. This finding is in accordance with studies showing transient fluctuations in gut bacterial profiles after introducing probiotic products or diet changes along with a rapid reversion of the microbial community to its previous stable state [46].

The final number of species/phylotypes detected in the present study was somewhat lower than that reported in other studies; however, the phylum, genus, and species representation follow the patterns reported in other studies that analyzed comparable samples and populations [54, 55]. The somewhat lower taxa numbers should be considered in light of the restrictive filtering criteria applied herein. Thus, only sequence clusters with at least 2 sequences and only clusters with a sequence identity of at least 98.5% with the HOMD sequences were taxonomically assigned. OTUs with identities of 97% to <98.5% could only be taxonomically assigned to the genus level and were not retained. The numbers of OTUs reported for saliva or tooth biofilm samples in different studies have greatly varied, which partly reflects biological or ethnic variations as well as variations in filtering criteria and conditions for taxonomical assignment [31]. In the present study, bacterial taxa were identified through alignment with the curated, chimera-free HOMD [2]. This database contains information on approximately 700 prokaryote species found in the human oral cavity; among these, approximately 49% taxa are named, 17% are cultivated but unnamed, and 34% are known only as uncultivated phylotypes. Regardless, mapping from a section of the 16S rRNA gene cannot separate closely related species, such as several members of the *Streptococcus* genus. For example, the relative prevalence rates of the 4 species in the most prevalent group (*S*. *oralis*, *S*. *mitis*, *S*. *mitis bv2*, *and S*. *infantis)* cannot be distinguished. Therefore, the species-level sequence abundance is partly due to the restrictive identification criteria and partly to the overall limitations of using 16S rRNA gene variation for species identification [56, 57].

To the best of our knowledge, this is the first study to evaluate the effects of a probiotic bacterium, *L*. *reuteri*, on the composition of tooth biofilm microbiota using 16S rRNA pyrosequencing. Multiplex sequencing is cost-effective for the following reasons: (*i*) thousands of sequences can be simultaneously obtained from a single sample [54], (*ii*) open-ended view of the microbiome is provided, and (*iii*) answers regarding microbial richness and ecological stability and shift are facilitated [58]. Drawbacks of this method include (*i*) limitations on taxonomic resolution due to the short-length sequence limit [58] *(ii*), loss of an unknown amount of taxa because of the rather high detection limit [59], and *(iii*) distortion of the true microbiota profile at various steps, i.e. contaminations in DNA extraction and purification, and PCR and sequencing errors and data analyses and interpretation [60, 61, 62]. Furthermore, Lagier *et al*. [59] specified the respective detection limits in large-scale molecular studies, such as pyrosequencing, conventional PCR, and cultivation as 10^6^, 10^4^, and 10^2^ CFU/mL, respectively. Those authors also reported that only 35% of bacterial phylotypes were detected in fecal samples by pyrosequencing. The fact that no *L*. *reuteri* or other lactobacilli (except two single sequences that were discarded according to the filtering criteria) was detected in 12-week samples from the test group, despite culture and PCR results demonstrating that the species and specifically the test strains were present in 6 of 7 test subjects, illustrates the underestimated number of detected species in the present study. Accordingly, metagenomic studies may need to consider this limitation as well as additional approaches for identifying less abundant, condition-associated genera/species. In contrast, the strengths of the study include the RCT design and high compliance and a double-blinded, randomized design in addition to the application of a new metagenomic approach combined with strain-specific PCR and cultivation.

Efforts to modify tooth biofilm and prevent oral diseases by consuming “beneficial bacteria” (probiotics) have been made for decades [63]. The fact that nearly one third of the test subjects in the present study did not present evidence of *L*. *reuteri* DSM 17938 and PTA 5289 strains, which is similar to findings from other studies with these strains or *L*. *rhamnosus* GG [33,34,64,65], indicates that the expected effect is individual. Installation of probiotic lactobacilli likely reflects genetic variations in host receptor epitopes for bacterial binding, such as salivary gp340, MUC5, MUC7, and ABO antigen epitopes [10,66–68].

The conclusion from the present study is that *L*. *reuteri* consumption induces a shift in oral microbiota composition but does not affect the total species richness. The biological relevance of this finding as well as the individuality of this effect remains to be elucidated.

## Supporting Information

S1 CONSORT Checklist CONSORT 2010 checklist of information. (PDF)

S1 Protocol Study protocol. (DOCX)

S1 Table Phylum and genus identities and abundances of the 257 identified taxa among the 45 included pyrosequencing samples. Mean abundances (% of all sequences) for test and control subjects at baseline and after 12 weeks exposure to an *L*. *reuteri* or placebo lozenge are listed.
(DOCX)

S2 Table Species and phylotypes identified by pyrosequencing of the 257 identified taxa among the 45 included samples. Mean abundances (% of all sequences) for test and control subjects at baseline and after 12 weeks exposure to an *L*. *reuteri* or placebo lozenge are listed.
(DOCX)
