# Supplementary material for: Oral Microbiota Shift after 12-Week Supplementation with Lactobacillus reuteri DSM 17938 and PTA 5289; A Randomized Control Trial
Source: PLoS One. 2015 May 6;10(5):e0125812. doi: 10.1371/journal.pone.0125812 (PMC4422650; doi:10.1371/journal.pone.0125812)
Supplement: S1 Protocol — (DOCX) [file pone.0125812.s002.docx]

**S1 PROTOCOL**

Metabolomics and genomics of tooth biofilms upon probiotics exposure (OMIC II)

**Background**

Dental caries is one of the most common chronic infectious diseases in the world [[Anusavice, 2002](#_ENREF_2)]. Despite the massive impact of dental caries on global welfare, the mechanisms of action and effective prevention and treatment in the susceptible fraction are insufficiently defined [[Lancet, 2009](#_ENREF_11)].

Research regarding probiotics to prevent oral diseases has increased in recent years. There is a concept where these “beneficial” microorganisms can inhibit a detrimental biofilm formation and protect oral tissue from disease [[Caglar et al., 2005](#_ENREF_4); [Meurman and Stamatova, 2007](#_ENREF_14)]. The most common probiotic strains belong to the genera *lactobacillus* and *bifidobacterium* [[Meurman and Stamatova, 2007](#_ENREF_14)]. Lactobacilli species have been associated with the development of dental caries because their aciduric and acidogenic traits [[Van Houte, 1980](#_ENREF_25)], however, they have also been linked to beneficial probiotic traits by mechanisms that include ecology modulation and immune stimulation [[McFarland, 2000](#_ENREF_13); [Mikelssar et al., 2004](#_ENREF_15); [Perdigón et al., 2001](#_ENREF_17)].

*L. reuteri* is a Gram-positive, non-sporeforming, non-motile, facultative anaerobic rod shaped bacillus [[Sinkiewicz, 2010](#_ENREF_22)] which produces a number of antibacterial compounds including, reuterin, a broad-spectrum antimicrobial structurally related to hydroxylpropionaldehyde [[Reuter, 2001](#_ENREF_18)]. Colonization of probiotic bacteria is considered an important event in establishing their beneficial effects. The mechanism of colonization/adherence in the gastrointestinal (GI) tract, i.e. “how” this occurs is unknown. However, colonization of the GI can be improved when *L. reuteri* is exposed to mucin [[Roos and Jonsson, 2002](#_ENREF_19)]. Long-term colonization by probiotic bacteria seems unlikely and bacteria has been only detected during the time probiotic products are in active use [[Haukioja, 2010](#_ENREF_6)].

We recently reported that *L. reuteri* strains DMS 17938 could colonize the oral cavity of 60-70% of subjects consuming them [Vestman *et al*., 2013]. In that study we also developed two selective media and presented specific primers for identification of *L. reuteri* DMS 17938 and ATCC PTA 5289.

**Aim**

In the present study, we attempt to *(i)* determine the profile of small molecules in supragingival plaque (teeth)*, and* *(ii)* to compare the oral microbiota between individuals with and without exposure of *L. reuteri* in a longitudinal RCT design and by use of pyrosequencing analysis.

**Hypothesis**

*L. reuteri* would influence biofilm ecology towards a less cariogenic microbiota and consequently showed variation in the metabolic profile.

**Methods**

**Study group**

Healthy volunteers will be recruited by advertising on boards at the Faculty of medicine, University of Umeå, Sweden. The inclusion criteria will be: *(i)* healthy with no reported medical condition and no active caries lesions or periodontitis. *(ii)* no use of antibiotics the last 3 months , *(iii)* no reported probiotic consumption 3 months prior to the study and *(iv)* no smoking. Based on previous knowledge of acquisition of probiotic bacteria, at least 40 individuals will be included in this study. Ethical approval will be obtained and all participants will be asked for a written consent at their baseline visit.

**Study design and intervention**

The study design is an explorative, double blinded, placebo-controlled randomized clinical trial with an exposure period of 12 weeks and 1- and 6- month follow-ups. Subjects will be randomly selected to a test or a control group using SPSS. Randomization will be done by a person that will not participate in sample collection. During the intervention period subjects will be asked to let one lozenge slowly melt in the mouth twice a day. The test lozenges contain *L. reuteri* (DSM 17938 and PTA 5289; 10^8^ CFU per strain; BioGaia AB, Stockholm, Sweden), isomalt, hydrogenated palm oil, peppermint and menthol flavoring, peppermint oil, and sucralose (<http://www.biogaia.com/product/biogaia-prodentis-oral-lozenges>). The safety of the lozenges has been documented and they are commercially available as an over the counter product. Except for the lactobacilli, the placebo lozenge are identical in composition, appearance and taste to the test lozenge.

**Microbiological Sampling**

Samples (saliva and dental biofilm) will be obtained at baseline and at 4, 8 and 12 weeks during *L. reuteri* exposure. Follow-up samples will be collected 1 and 6 months after finishing the exposure period. Stimulated saliva (~5mL) will be collected into ice chilled sterile test tubes during chewing on paraffin, and ~1mL of saliva will be used for cultivation on selective and not selective agar plates in our laboratory. The remaining saliva will be centrifuged and stored at -80ºC (pellets and supernatant separately) for pyrosequencing analysis. Lozenges extracts will also be cultivated to control presence/absence of lactobacilli, and grown lactobacilli would be identified by species/strain specific culturing combined with 16S rDNA PCR amplification and sequencing. Biofilm will be sampled with sterile tooth-picks from available smooth tooth surfaces in the premolar/molar and upper incisor regions. Pooled biofilm will be divided into two portions, one for metabolomics and one in TE-buffer for DNA extraction for the pyrosequencing. Samples will be stored in sterile Eppendorf tubes at -80°C until analysed.

**GC-TOFMS metabolomics for fingerprinting of small molecules**

The protocol for GC-TOFMS follows [Wuolikainen et al. [2011](#_ENREF_27)]. Thus, low molecular weight compounds/metabolites are extracted from tooth biofilm samples with methanol/water supplied with labeled internal standards. The extracts are derivatized by addition of *O*-methylhydroxylamine hydrochloride in pyridine and an injection standard (methylstearate) is added. For the GC-TOFMS analysis 1 μL aliquot is injected splitless into a gas chromatograph equipped with a 10 m fused-silica capillary column chemically bonded with 0.18 μm DB 5-MS stationary phase. The injector temperature is set to 270°C and the septum purge gas is turned on after 60 s at a flow rate of 20 mL/min. The initial column temperature is 70 °C for 2 min, followed by increases by 40°C /min until a temperature of 320°C is reached. The oven is then allowed to return to and stabilize at the starting temperature. The gas flow rate through the column is 1 mL/min. The column outlet is connected to the ion source of a Pegasus III TOFMS. Transfer line temperature is set to 250°C and ion source temperature to 200°C. Ions are produced by a 70 eV electron beam (2.0 mA). Acceleration voltage is switched on after a 165 s solvent delay and masses are acquired between *m/z* 50-800, at a rate of 30 spectra s-1 for 445 s.

Settings for resolving chromatographic profiles are rated by maximum number of resolved profiles, minimum number of bad spectra, splitting of internal standard peaks and library hit of internal standards. Detected metabolites are identified by chromatographic retention times, and resolved mass spectra are compared against in-house and publicly available mass spectra libraries (NIST 98 mass spectra library, the Umeå Plant Science Centre mass spectra library and the mass spectra library hosted by the Max Planck Institute) or *de novo* identification. Unknown metabolites will (if possible) be categorized into chemical classes, such as fatty acid, amino acid, etc. Data will be normalized to the 11 internal standards and the injection standard.

**Pyrosequencing for detection of the complex microbiota**

Biofilm samples will be analyzed with 454 pyrosequencing either the bench top GS Junior machine or FLX+ with titanium chemistry (Roche). This should yield between 100 000 to 1 000 000 reads with a sequence mean length of at least 400 basepairs (bp). Ligation of a sample-specific barcodes allow several samples to be run simultaneously. There are four major steps in pyrosequencing. (1) library construction, including DNA extraction, purification and concentration, 16S rDNA amplification using universal primers amplifying a 600-bp segment in the V3-V4 hypervariable region of the 16S rRNA gene, ligation of 21 bp 3’ and a 5’ titanium adaptor sequences, and the barcode for sample origin recognition. The library may be stored at-20°C for months. (2) Emulsion amplification where capture beads carrying one ligated PCR reagent/bead is placed into a water in oil microreactor (droplet), in which amplification takes place (using universal primers to the adaptor sequences). (3) Sequencing of fragments on DNA positive beads placed in nano-wells on a slide after breaking the microreactors and enrichment of amplicon carrying beads. (4) Data processing include trimming, quality filtering and chimera removal using the QIIME pipeline. Sequences will be clustered into OTUs at 97% similarity against the Greengene database and taxonomically identified against the HOMD database. Identified species/phylotypes will be further analyzed by traditional univariate analyses for means and proportions and multivariate projection methods.

**Statistical power**

Previous studies using probiotic products have shown statistically significant effects on mutans streptococci in groups of 20 subjects with high levels of mutans streptococci. We will recruit at least 40 subjects to ensure that we have a sufficient number of subjecta were the test bacteria is retained during exposure and to get subjects with different levels of mutans streptococci to facilitate effects in strata of mutans streptococci levels, and to account for that some subjects for some reason will terminate their participation.

Data will be analyzed using SIMCA 12+ and SAS depending on character of the analysis. For description means (95%CI) and proportion distributions will be calculated and differences between groups will be tested with parametric or non-parametric tests depending on data character and distribution. For most parts of the analyses multiple comparison adjustments by the false discovery rate (recommended for large number of measures such as in genome wide arrays) will be applied. Multivariate analyses will be done with PCA, PLS(-DA), and OPLS-(DA). Prior to multivariate data analysis GC-TOFMS internal standards and methylstearate are excluded and all data are mean centred and scaled to unit variance. This method has been very useful for datasets where the number of variables exceeds the number of cases and where variables co-vary [[Kanasi et al., 2010](#_ENREF_10)].

**Significance of the study**

This study will confirm or reject *in vivo* biofilm modifications due to exposure to probiotic bacteria. In contrast to previous studies focusing on single microorganisms, such as mutans streptococci and candida, and single events, such as plaque pH, the present study will give a simultaneous fingerprint of the biological molecule and microbiota complex.

This study may also introduce/strengthen in house knowledge of front line methods for high-throughput, in-depth characterization of environmental and bacterial dynamics in the biofilm*.* Access to such tools allows studies to move from focusing on a few disease-associated components/species, such as single proteins or mutans streptococci, to a simultaneous imprint of the complex *in vivo* conditions.

References

Abrahamsson TR, Jakobsson T, Böttcher MF, Fredrikson M, Jenmalm MC, Björkstén B, Oldaeus G: Probiotics in prevention of ige-associated eczema: A double-blind, randomized, placebo-controlled trial. Journal of Allergy and Clinical Immunology 2007;119:1174-1180.

Anusavice KJ: Dental caries: Risk assessment and treatment solutions for an elderly population. Compend Contin Educ Dent 2002;23:12-20.

Caglar E, Cildir SK, Ergeneli S, Sandalli N, Twetman S: Salivary mutans streptococci and lactobacilli levels after ingestion of the probiotic bacterium lactobacillus reuteri atcc 55730 by straws or tablets. Acta odontologica Scandinavica 2006;64:314-318.

Caglar E, Kargul B, Tanboga I: Bacteriotherapy and probiotics' role on oral health. Oral diseases 2005;11:131-137.

Hasslof P, Hedberg M, Twetman S, Stecksen-Blicks C: Growth inhibition of oral mutans streptococci and candida by commercial probiotic lactobacilli--an in vitro study. BMC oral health 2010;10:18.

Haukioja A: Probiotics and oral health. European journal of dentistry 2010;4:348-355.

Haukioja A, Yli-Knuuttila H, Loimaranta V, Kari K, Ouwehand AC, Meurman JH, Tenovuo J: Oral adhesion and survival of probiotic and other lactobacilli and bifidobacteria in vitro. Oral Microbiol Immunol 2006;21:326-332.

Holgerson PL, Vestman NR, Claesson R, Ohman C, Domellof M, Tanner AC, Hernell O, Johansson I: Oral microbial profile discriminates breastfed from formula-fed infants. Journal of pediatric gastroenterology and nutrition 2012.

Imase K, Tanaka A, Tokunaga K, Sugano H, Ishida H, Takahashi S: Lactobacillus reuteri tablets suppress helicobacter pylori infection-a double-blind randomised placebo-controlled cross-over clinical study. JOURNAL-JAPANESE ASSOCIATION FOR INFECTIOUS DISEASES 2007;81:387.

Kanasi E, Johansson I, Lu SC, Kressin NR, Nunn ME, Kent R, Jr., Tanner AC: Microbial risk markers for childhood caries in pediatricians' offices. Journal of dental research 2010;89:378-383.

Lancet: Oral health: Prevention is key. The Lancet 2009;373:1.

Mayr M: Recent highlights of metabolomics in cardiovascular research. Circulation Cardiovascular genetics 2011;4:463-464.

McFarland LV: Normal flora: Diversity and functions. Microbial ecology in health and disease 2000;12:193-207.

Meurman JH, Stamatova I: Probiotics: Contributions to oral health. Oral diseases 2007;13:443-451.

Mikelssar M, Mandar R, Sepp E, Annuk H: Human lactic acid microﬂora and its role in the welfare of the host; in: Lactic acid bacteria: microbiological and functional aspects. 2004, vol 139, p 453.

Nikawa H, Makihira S, Fukushima H, Nishimura H, Ozaki Y, Ishida K, Darmawan S, Hamada T, Hara K, Matsumoto A, Takemoto T, Aimi R: Lactobacillus reuteri in bovine milk fermented decreases the oral carriage of mutans streptococci. International journal of food microbiology 2004;95:219-223.

Perdigón G, Fuller R, Raya R: Lactic acid bacteria and their effect on the immune system. Current issues in intestinal microbiology 2001;2:27-42.

Reuter G: The lactobacillus and bifidobacterium microflora of the human intestine: Composition and succession. Curr Issues Intest Microbiol 2001;2:43-53.

Romani Vestman N, Hasslof P, Keller M, Roos S, Twetman S, et al. (2013) Persistence of L. reuteri and its effects on salivary mutans streptococci.

Roos S, Jonsson H: A high-molecular-mass cell-surface protein from lactobacillus reuteri 1063 adheres to mucus components. Microbiology 2002;148:433-442.

Saavedra J: Probiotics and infectious diarrhea. The American journal of gastroenterology 2000;95:S16-S18.

Savino F: Focus on infantile colic. Acta Paediatrica 2007;96:1259-1264.

Sinkiewicz G: Lactobacillus reuteri in health and disease. 2010.

Takahashi N, Washio J, Mayanagi G: Metabolomics of supragingival plaque and oral bacteria. Journal of dental research 2010;89:1383-1388.

Thysell E, Surowiec I, Hornberg E, Crnalic S, Widmark A, Johansson AI, Stattin P, Bergh A, Moritz T, Antti H, Wikstrom P: Metabolomic characterization of human prostate cancer bone metastases reveals increased levels of cholesterol. PloS one 2010;5:e14175.

Van Houte J: Bacterial specificity in the etiology of dental caries. International dental journal 1980;30:305.

Wei J, Xie G, Zhou Z, Shi P, Qiu Y, Zheng X, Chen T, Su M, Zhao A, Jia W: Salivary metabolite signatures of oral cancer and leukoplakia. International journal of cancer Journal international du cancer 2011;129:2207-2217.

Wuolikainen A, Moritz T, Marklund SL, Antti H, Andersen PM: Disease-related changes in the cerebrospinal fluid metabolome in amyotrophic lateral sclerosis detected by gc/tofms. PloS one 2011;6:e17947.
