# Supplementary material for: Oral Microbiota Shift after 12-Week Supplementation with Lactobacillus reuteri DSM 17938 and PTA 5289; A Randomized Control Trial
Source: PLoS One. 2015 May 6;10(5):e0125812. doi: 10.1371/journal.pone.0125812 (PMC4422650; doi:10.1371/journal.pone.0125812)
Supplement: S1 Table — Mean abundances (% of all sequences) for test and control subjects at baseline and after 12 weeks exposure to an L. reuteri or placebo lozenge are listed. (DOCX) [file pone.0125812.s003.docx]

**S1 Table. Phylum and genus identities and abundances of the 257 identified taxa among the 45 included pyrosequencing samples.** Mean abundances (% of all sequences) for test and control subjects at baseline and after 12 weeks exposure to an *L. reuteri* or placebo lozenge are listed.

|  |  | **Phylum prevalence (%)** | | | | |  | **Genus prevalence (%)** | | | | |
| --- | --- | --- | --- | --- | --- | --- | --- | --- | --- | --- | --- | --- |
|  |  | **test group** | |  | **control group** | |  | **test group** | |  | **placebo group** | |
|  |  |  | **12** |  |  | **12** |  |  | **12** |  |  | **12** |
| **Phylum** | **Genus** | **baseline** | **weeks** |  | **baseline** | **weeks** |  | **baseline** | **weeks** |  | **baseline** | **weeks** |
| Actinobacteria |  | 1.695 | 2.251 |  | 5.129 | 5.181 |  |  |  |  |  |  |
|  | Actinobaculum |  |  |  |  |  |  | 0.006 | 0.003 |  | 0.014 | 0.003 |
|  | Actinomyces |  |  |  |  |  |  | 1.345 | 1.806 |  | 4.595 | 4.465 |
|  | Atopobium |  |  |  |  |  |  | 0.192 | 0.008 |  | 0.025 | 0.008 |
|  | Corynebacterium |  |  |  |  |  |  | 0.015 | 0.106 |  | 0.004 | 0.013 |
|  | Rothia |  |  |  |  |  |  | 0.130 | 0.321 |  | 0.491 | 0.691 |
|  | Scardovia |  |  |  |  |  |  | 0.000 | 0.008 |  | 0.000 | 0.000 |
|  | Slackia |  |  |  |  |  |  | 0.006 | 0.000 |  | 0.000 | 0.000 |
| Bacteroidetes |  | 13.913 | 8.035 |  | 12.515 | 11.602 |  |  |  |  |  |  |
|  | Alloprevotella |  |  |  |  |  |  | 0.589 | 0.599 |  | 0.637 | 1.273 |
|  | Bacteroidales[G-2] |  |  |  |  |  |  | 0.062 | 0.008 |  | 0.053 | 0.065 |
|  | Bacteroidetes[G-5] |  |  |  |  |  |  | 0.000 | 0.000 |  | 0.009 | 0.011 |
|  | Bergeyella |  |  |  |  |  |  | 0.186 | 0.371 |  | 0.158 | 0.283 |
|  | Capnocytophaga |  |  |  |  |  |  | 5.697 | 2.893 |  | 3.478 | 3.835 |
|  | Porphyromonas |  |  |  |  |  |  | 3.285 | 2.416 |  | 3.962 | 3.114 |
|  | Prevotella |  |  |  |  |  |  | 4.029 | 1.657 |  | 3.875 | 2.875 |
|  | Tannerella |  |  |  |  |  |  | 0.065 | 0.090 |  | 0.342 | 0.090 |
| Firmicutes |  | 72.342 | 74.256 |  | 60.754 | 64.303 |  |  |  |  |  |  |
|  | Abiotrophia |  |  |  |  |  |  | 9.075 | 3.386 |  | 1.280 | 2.434 |
|  | Catonella |  |  |  |  |  |  | 0.003 | 0.003 |  | 0.005 | 0.019 |
|  | Centipeda |  |  |  |  |  |  | 0.006 | 0.013 |  | 0.016 | 0.073 |
|  | Clostridiales[F-2][G-1] |  |  |  |  |  |  | 0.009 | 0.093 |  | 0.042 | 0.087 |
|  | Clostridiales[F-2][G-2] |  |  |  |  |  |  | 0.003 | 0.005 |  | 0.004 | 0.021 |
|  | Dialister |  |  |  |  |  |  | 0.019 | 0.013 |  | 0.011 | 0.040 |
|  | Eubacterium[11][G-3] |  |  |  |  |  |  | 0.040 | 0.027 |  | 0.062 | 0.031 |
|  | Eubacterium[11][G-7] |  |  |  |  |  |  | 0.003 | 0.000 |  | 0.009 | 0.031 |
|  | Filifactor |  |  |  |  |  |  | 0.009 | 0.000 |  | 0.004 | 0.024 |
|  | Gemella |  |  |  |  |  |  | 3.964 | 4.596 |  | 2.098 | 2.854 |
|  | Granulicatella |  |  |  |  |  |  | 2.055 | 4.187 |  | 2.147 | 2.460 |
|  | Johnsonella |  |  |  |  |  |  | 0.000 | 0.011 |  | 0.020 | 0.048 |
|  | Lachnoanaerobaculum |  |  |  |  |  |  | 0.267 | 0.178 |  | 1.023 | 1.044 |
|  | Lachnospiraceae[G-2] |  |  |  |  |  |  | 0.009 | 0.011 |  | 0.005 | 0.258 |
|  | Lachnospiraceae[G-3] |  |  |  |  |  |  | 0.077 | 0.117 |  | 0.328 | 0.152 |
|  | Lachnospiraceae[G-5] |  |  |  |  |  |  | 0.000 | 0.000 |  | 0.000 | 0.011 |
|  | Megasphaera |  |  |  |  |  |  | 0.003 | 0.000 |  | 0.031 | 0.002 |
|  | Mitsuokella |  |  |  |  |  |  | 0.000 | 0.000 |  | 0.034 | 0.002 |
|  | Mogibacterium |  |  |  |  |  |  | 0.015 | 0.016 |  | 0.014 | 0.000 |
|  | Moryella |  |  |  |  |  |  | 0.121 | 0.040 |  | 0.208 | 0.223 |
|  | Oribacterium |  |  |  |  |  |  | 0.000 | 0.000 |  | 0.004 | 0.031 |
|  | Parvimonas |  |  |  |  |  |  | 0.183 | 0.122 |  | 0.089 | 0.079 |
|  | Peptococcus |  |  |  |  |  |  | 0.012 | 0.005 |  | 0.036 | 0.090 |
|  | Peptostreptococcaceae[11][G-2] |  |  |  |  |  |  | 0.000 | 0.000 |  | 0.000 | 0.003 |
|  | Peptostreptococcaceae[11][G-4] |  |  |  |  |  |  | 0.000 | 0.000 |  | 0.000 | 0.010 |
|  | Peptostreptococcaceae[11][G-5] |  |  |  |  |  |  | 0.000 | 0.000 |  | 0.000 | 0.008 |
|  | Peptostreptococcaceae[11][G-7] |  |  |  |  |  |  | 0.000 | 0.000 |  | 0.000 | 0.006 |
|  | Peptostreptococcus |  |  |  |  |  |  | 0.034 | 0.016 |  | 0.036 | 0.073 |
|  | Selenomonas |  |  |  |  |  |  | 3.502 | 2.652 |  | 4.785 | 3.827 |
|  | Shuttleworthia |  |  |  |  |  |  | 0.003 | 0.000 |  | 0.011 | 0.000 |
|  | Streptococcus |  |  |  |  |  |  | 47.355 | 55.285 |  | 43.463 | 43.087 |
|  | Veillonella |  |  |  |  |  |  | 5.501 | 3.466 |  | 4.970 | 7.266 |
|  | Veillonellaceae[G-1] |  |  |  |  |  |  | 0.071 | 0.016 |  | 0.018 | 3.735 |
| Fusobacteria |  | 5.167 | 6.274 |  | 12.386 | 10.805 |  |  |  |  |  |  |
|  | Fusobacterium |  |  |  |  |  |  | 3.694 | 2.564 |  | 6.766 | 4.683 |
|  | Leptotrichia |  |  |  |  |  |  | 1.472 | 3.710 |  | 5.620 | 6.122 |
| Proteobacteria |  | 6.722 | 8.937 |  | 8.915 | 7.646 |  |  |  |  |  |  |
|  | Aggregatibacter |  |  |  |  |  |  | 0.487 | 3.498 |  | 1.135 | 2.567 |
|  | Campylobacter |  |  |  |  |  |  | 0.325 | 0.448 |  | 1.021 | 0.735 |
|  | Cardiobacterium |  |  |  |  |  |  | 0.022 | 0.101 |  | 0.042 | 0.082 |
|  | Eikenella |  |  |  |  |  |  | 0.015 | 0.029 |  | 0.029 | 0.048 |
|  | Haemophilus |  |  |  |  |  |  | 4.249 | 2.898 |  | 5.531 | 1.917 |
|  | Kingella |  |  |  |  |  |  | 0.139 | 0.082 |  | 0.176 | 0.300 |
|  | Lautropia |  |  |  |  |  |  | 0.093 | 0.284 |  | 0.210 | 0.200 |
|  | Neisseria |  |  |  |  |  |  | 1.392 | 1.591 |  | 0.746 | 1.696 |
|  | Ottowia |  |  |  |  |  |  | 0.000 | 0.005 |  | 0.025 | 0.099 |
| SR1 | SR1[G-1] | 0.099 | 0.180 |  | 0.206 | 0.391 |  | 0.099 | 0.180 |  | 0.206 | 0.391 |
| Synergistetes | Fretibacterium | 0.015 | 0.037 |  | 0.005 | 0.023 |  | 0.015 | 0.037 |  | 0.005 | 0.023 |
| Tenericutes | Mycoplasma | 0.000 | 0.000 |  | 0.000 | 0.013 |  | 0.000 | 0.000 |  | 0.000 | 0.008 |
| TM7 |  | 0.046 | 0.029 |  | 0.089 | 0.042 |  |  |  |  |  |  |
|  | TM7[G-1] |  |  |  |  |  |  | 0.009 | 0.011 |  | 0.027 | 0.027 |
|  | TM7[G-2] |  |  |  |  |  |  | 0.000 | 0.000 |  | 0.007 | 0.000 |
|  | TM7[G-3] |  |  |  |  |  |  | 0.003 | 0.005 |  | 0.049 | 0.002 |
|  | TM7[G-4] |  |  |  |  |  |  | 0.034 | 0.013 |  | 0.005 | 0.013 |
