# Supplementary material for: Oral Microbiota Shift after 12-Week Supplementation with Lactobacillus reuteri DSM 17938 and PTA 5289; A Randomized Control Trial
Source: PLoS One. 2015 May 6;10(5):e0125812. doi: 10.1371/journal.pone.0125812 (PMC4422650; doi:10.1371/journal.pone.0125812)
Supplement: S2 Table — Mean abundances (% of all sequences) for test and control subjects at baseline and after 12 weeks exposure to an L. reuteri or placebo lozenge are listed. (DOCX) [file pone.0125812.s004.docx]

**S2 Table. Species and phylotypes identified by pyrosequencing of the 257 identified taxa among the 45 included samples.** Mean abundances (% of all sequences) for test and control subjects at baseline and after 12 weeks exposure to an *L. reuteri* or placebo lozenge are listed.

|  |  | **Species/phylotype prevalence (%)** | | | | |
| --- | --- | --- | --- | --- | --- | --- |
|  |  | **test group** | |  | **placebo group** | |
| **Genus** | **HOMD clone name** | **baseline** | **12 weeks** |  | **baseline** | **12 weeks** |
| Actinobaculum | *Actinobaculum sp.* HOT183 | 0.010 | 0.004 |  | 0.014 | 0.003 |
| Actinomyces | *Actinomyces georgiae* | 0.000 | 0.000 |  | 0.006 | 0.006 |
|  | *Actinomyces gerencseriae* | 0.026 | 0.031 |  | 0.057 | 0.010 |
|  | *Actinomyces israelii* | 0.000 | 0.000 |  | 0.004 | 0.002 |
|  | *Actinomyces johnsonii* | 0.116 | 0.244 |  | 0.170 | 0.333 |
|  | *Actinomyces massiliensis* | 0.192 | 0.195 |  | 0.464 | 0.557 |
|  | *Actinomyces meyeri* | 0.007 | 0.003 |  | 0.074 | 0.080 |
|  | *Actinomyces naeslundii* | 0.394 | 0.403 |  | 0.705 | 0.615 |
|  | *Actinomyces oris* | 0.125 | 0.175 |  | 0.953 | 1.188 |
|  | *Actinomyces sp.* HOT170 | 0.046 | 0.159 |  | 0.062 | 0.083 |
|  | *Actinomyces sp.* HOT171 | 0.062 | 0.141 |  | 0.180 | 0.397 |
|  | *Actinomyces sp. HOT175* | 0.008 | 0.012 |  | 0.110 | 0.052 |
|  | *Actinomyces sp.* HOT177 | 0.183 | 0.260 |  | 0.519 | 0.584 |
|  | *Actinomyces sp.* HOT178 | 0.019 | 0.022 |  | 0.050 | 0.026 |
|  | *Actinomyces sp.* HOT180 | 0.190 | 0.191 |  | 0.479 | 0.538 |
|  | *Actinomyces sp.* HOT448 | 0.000 | 0.000 |  | 0.003 | 0.000 |
|  | *Actinomyces sp.* HOT877 | 0.006 | 0.000 |  | 0.013 | 0.009 |
|  | *Actinomyces sp.* HOT848 | 0.000 | 0.000 |  | 0.001 | 0.003 |
| Atopobium | *Atopobium parvulum* | 0.019 | 0.000 |  | 0.011 | 0.004 |
|  | *Atopobium rimae, A. sp.* HOT199 | 0.114 | 0.010 |  | 0.009 | 0.005 |
| Corynebacterium | *Corynebacterium durum* | 0.005 | 0.037 |  | 0.000 | 0.007 |
|  | *Corynebacterium matruchotii* | 0.011 | 0.047 |  | 0.003 | 0.011 |
| Rothia | *Rothia aeria* | 0.058 | 0.166 |  | 0.101 | 0.129 |
|  | *Rothia dentocariosa* | 0.051 | 0.085 |  | 0.455 | 1.048 |
| Scardovia | *Scardovia wiggsiae* | 0.000 | 0.008 |  | 0.000 | 0.000 |
| Slackia | *Slackia exigua* | 0.004 | 0.000 |  | 0.000 | 0.000 |
| Alloprevotella | *Alloprevotella rava* | 0.000 | 0.000 |  | 0.003 | 0.000 |
|  | *Alloprevotella sp.* HOT308 | 0.017 | 0.015 |  | 0.002 | 0.011 |
|  | *Alloprevotella sp.* HOT473 | 0.091 | 0.425 |  | 0.373 | 0.676 |
|  | *Alloprevotella sp.* HOT912 | 0.011 | 0.049 |  | 0.337 | 0.301 |
|  | *Alloprevotella sp.* HOT914 | 0.069 | 0.032 |  | 0.067 | 0.102 |
|  | *Alloprevotella tannerae* | 0.264 | 0.170 |  | 0.012 | 0.045 |
| Bacteroidales[G-2] | *Bacteroidales [G-2] sp.* HOT274 | 0.048 | 0.008 |  | 0.038 | 0.061 |
| Bacteroidetes[G-5] | *Bacteroidetes [G-5] sp.* HOT505 | 0.000 | 0.000 |  | 0.014 | 0.009 |
|  | *Bacteroidetes [G-5] sp.* HOT511 | 0.000 | 0.000 |  | 0.003 | 0.051 |
| Bergeyella | *Bergeyella sp. HOT322* | 0.195 | 0.354 |  | 0.170 | 0.295 |
|  | *Bergeyella sp. HOT900* | 0.002 | 0.000 |  | 0.006 | 0.003 |
|  | *Bergeyella sp. HOT907* | 0.000 | 0.002 |  | 0.003 | 0.006 |
| Capnocytophaga | *Capnocytophaga gingivalis* | 0.411 | 0.414 |  | 0.872 | 0.332 |
|  | *Capnocytophaga granulosa* | 0.114 | 0.051 |  | 0.125 | 0.172 |
|  | *Capnocytophaga haemolytica* | 0.000 | 0.000 |  | 0.011 | 0.004 |
|  | *Capnocytophaga leadbetteri* | 0.739 | 1.291 |  | 1.189 | 1.266 |
|  | *Capnocytophaga ochracea* | 0.007 | 0.000 |  | 0.001 | 0.007 |
|  | *Capnocytophaga sp.* HOT323 | 0.012 | 0.005 |  | 0.018 | 0.033 |
|  | *Capnocytophaga sp.* HOT324 | 0.000 | 0.000 |  | 0.034 | 0.027 |
|  | *Capnocytophaga sp.* HOT326 | 0.095 | 0.069 |  | 0.177 | 0.058 |
|  | *Capnocytophaga sp.* HOT332 | 0.035 | 0.064 |  | 0.015 | 0.009 |
|  | *Capnocytophaga sp.* HOT335 | 0.000 | 0.000 |  | 0.033 | 0.000 |
|  | *Capnocytophaga sp.* HOT336 | 0.016 | 0.020 |  | 0.008 | 0.003 |
|  | *Capnocytophaga sp.* HOT338 | 0.007 | 0.010 |  | 0.014 | 0.016 |
|  | *Capnocytophaga sp.* HOT412, HOT323 | 0.056 | 0.002 |  | 0.001 | 0.021 |
|  | *Capnocytophaga sp.* HOT863 | 0.000 | 0.009 |  | 0.003 | 0.000 |
|  | *Capnocytophaga sp.* HOT864 | 0.027 | 0.006 |  | 0.160 | 0.029 |
|  | *Capnocytophaga sp.* HOT878 | 0.000 | 0.000 |  | 0.005 | 0.000 |
|  | *Capnocytophaga sp.* HOT901 | 0.000 | 0.000 |  | 0.000 | 0.037 |
|  | *Capnocytophaga sp.* HOT902, HOT864, HOT412, HOT336 | 0.060 | 0.038 |  | 0.130 | 0.045 |
|  | *Capnocytophaga sp.* HOT903 | 0.000 | 0.007 |  | 0.037 | 0.031 |
|  | *Capnocytophaga sputigena* | 3.416 | 1.109 |  | 1.043 | 1.394 |
| Porphyromonas | *Porphyromonas catoniae* | 0.596 | 0.323 |  | 0.838 | 0.426 |
|  | *Porphyromonas endodontalis* | 0.149 | 0.102 |  | 0.022 | 0.139 |
|  | *Porphyromonas sp.* HOT275 | 0.000 | 0.000 |  | 0.003 | 0.002 |
|  | *Porphyromonas sp.* HOT277 | 0.017 | 0.017 |  | 0.022 | 0.481 |
|  | *Porphyromonas sp.* HOT278, HOT277 | 0.039 | 0.004 |  | 0.037 | 0.154 |
|  | *Porphyromonas sp.* HOT279 | 2.258 | 1.724 |  | 1.916 | 1.276 |
|  | *Porphyromonas sp.* HOT284, *P. catoniae* | 0.084 | 0.139 |  | 0.636 | 0.403 |
|  | *Porphyromonas sp.* HOT395 | 0.014 | 0.034 |  | 0.000 | 0.001 |
| Prevotella | *Prevotella buccae* | 0.002 | 0.000 |  | 0.000 | 0.002 |
|  | *Prevotella denticola* | 0.034 | 0.079 |  | 0.027 | 0.029 |
|  | *Prevotella fusca* | 0.000 | 0.000 |  | 0.003 | 0.004 |
|  | *Prevotella histicola* | 0.016 | 0.016 |  | 0.000 | 0.011 |
|  | *Prevotella intermedia* | 0.531 | 0.177 |  | 0.358 | 0.546 |
|  | *Prevotella maculosa* | 0.061 | 0.003 |  | 0.071 | 0.032 |
|  | *Prevotella marshii* | 0.005 | 0.000 |  | 0.000 | 0.008 |
|  | *Prevotella melaninogenica* | 0.700 | 0.675 |  | 0.285 | 0.422 |
|  | *Prevotella micans* | 0.005 | 0.021 |  | 0.016 | 0.025 |
|  | *Prevotella multiformis* | 0.010 | 0.003 |  | 0.016 | 0.000 |
|  | *Prevotella nigrescens* | 0.970 | 0.166 |  | 0.458 | 0.135 |
|  | *Prevotella oralis* | 0.000 | 0.000 |  | 0.000 | 0.003 |
|  | *Prevotella oris* | 0.251 | 0.043 |  | 0.135 | 0.140 |
|  | *Prevotella oulorum* | 0.055 | 0.062 |  | 0.353 | 0.374 |
|  | *Prevotella pallens, P. sp.* HOT310 | 0.017 | 0.009 |  | 0.019 | 0.008 |
|  | *Prevotella pleuritidis [NV]* | 0.007 | 0.014 |  | 0.000 | 0.006 |
|  | *Prevotella saccharolytica* | 0.030 | 0.046 |  | 0.114 | 0.155 |
|  | *Prevotella salivae* | 0.010 | 0.032 |  | 0.020 | 0.055 |
|  | *Prevotella sp.* HOT292 | 0.044 | 0.000 |  | 0.004 | 0.002 |
|  | *Prevotella sp.* HOT299 | 0.068 | 0.055 |  | 0.028 | 0.056 |
|  | *Prevotella sp.* HOT300 | 0.160 | 0.010 |  | 0.032 | 0.004 |
|  | *Prevotella sp.* HOT301 | 0.000 | 0.000 |  | 0.000 | 0.016 |
|  | *Prevotella sp.* HOT309 | 0.000 | 0.000 |  | 0.000 | 0.019 |
|  | *Prevotella sp.* HOT310 | 0.039 | 0.002 |  | 0.000 | 0.000 |
|  | *Prevotella sp.* HOT313 | 0.292 | 0.041 |  | 0.000 | 0.000 |
|  | *Prevotella sp.* HOT314 | 0.025 | 0.003 |  | 0.071 | 0.031 |
|  | *Prevotella sp.* HOT315 | 0.002 | 0.002 |  | 0.000 | 0.012 |
|  | *Prevotella sp.* HOT317 | 0.172 | 0.153 |  | 0.654 | 0.055 |
|  | *Prevotella sp.* HOT472 | 0.172 | 0.144 |  | 0.416 | 0.336 |
|  | *Prevotella sp.* HOT475 | 0.022 | 0.012 |  | 0.130 | 0.075 |
|  | *Prevotella veroralis* | 0.017 | 0.000 |  | 0.109 | 0.032 |
| Tannerella | *Tannerella forsythia* | 0.000 | 0.003 |  | 0.000 | 0.002 |
|  | *Tannerella sp.* HOT286 | 0.065 | 0.107 |  | 0.274 | 0.083 |
|  | *Tannerella sp.* HOT808 | 0.010 | 0.000 |  | 0.009 | 0.002 |
| Abiotrophia | *Abiotrophia defectiva* | 10.626 | 3.158 |  | 1.389 | 2.224 |
| Catonella | *Catonella morbi* | 0.002 | 0.002 |  | 0.008 | 0.015 |
|  | *Catonella sp. HOT451* | 0.000 | 0.000 |  | 0.000 | 0.003 |
| Centipeda | *Centipeda periodontii* | 0.007 | 0.017 |  | 0.014 | 0.079 |
| Clostridiales[F-2][G-1] | *Clostridiales [F-2][G-1] sp.* HOT075 | 0.007 | 0.070 |  | 0.040 | 0.091 |
| Clostridiales[F-2][G-2] | *Clostridiales [F-2][G-2] sp.* HOT085 | 0.002 | 0.004 |  | 0.004 | 0.017 |
| Dialister | *Dialister invisus* | 0.008 | 0.017 |  | 0.001 | 0.000 |
|  | *Dialister pneumosintes* | 0.005 | 0.000 |  | 0.012 | 0.038 |
| Eubacterium[11][G-3] | *Eubacterium [XI][G-3] brachy* | 0.028 | 0.030 |  | 0.046 | 0.029 |
| Eubacterium[11][G-7] | *Eubacterium [XI][G-7] yurii* | 0.002 | 0.000 |  | 0.006 | 0.028 |
| Filifactor | *Filifactor alocis* | 0.007 | 0.000 |  | 0.006 | 0.021 |
| Gemella | *Gemella haemolysans* | 1.490 | 1.719 |  | 0.321 | 1.064 |
|  | *Gemella morbillorum* | 2.462 | 2.685 |  | 1.202 | 1.405 |
|  | *Gemella sanguinis* | 0.179 | 0.160 |  | 0.246 | 0.165 |
| Granulicatella | *Granulicatella adiacens* | 1.712 | 3.971 |  | 1.888 | 1.802 |
|  | *Granulicatella elegans* | 0.262 | 0.561 |  | 0.330 | 0.649 |
| Johnsonella | *Johnsonella ignava* | 0.000 | 0.009 |  | 0.024 | 0.029 |
|  | *Johnsonella sp. HOT166* | 0.000 | 0.000 |  | 0.000 | 0.013 |
| Lachnoanaerobaculum | *Lachnoanaerobaculum orale* | 0.039 | 0.006 |  | 0.059 | 0.376 |
|  | *Lachnoanaerobaculum saburreum* | 0.050 | 0.045 |  | 0.373 | 0.123 |
|  | *Lachnoanaerobaculum sp.* HOT083 | 0.000 | 0.000 |  | 0.000 | 0.009 |
|  | *Lachnoanaerobaculum umeaense* | 0.200 | 0.144 |  | 0.399 | 0.389 |
| Lachnospiraceae[G-2] | *Lachnospiraceae [G-2] sp.* HOT088 | 0.007 | 0.008 |  | 0.005 | 0.245 |
| Lachnospiraceae[G-3] | *Lachnospiraceae [G-3] sp.* HOT100 | 0.091 | 0.108 |  | 0.284 | 0.159 |
| Lachnospiraceae[G-5] | *Lachnospiraceae [G-5] sp.* HOT080 | 0.000 | 0.000 |  | 0.000 | 0.010 |
| Megasphaera | *Megasphaera micronuciformis* | 0.005 | 0.000 |  | 0.021 | 0.002 |
| Mitsuokella | *Mitsuokella sp.* HOT521 | 0.000 | 0.000 |  | 0.026 | 0.002 |
| Mogibacterium | *Mogibacterium neglectum, M. vescum* | 0.010 | 0.020 |  | 0.010 | 0.000 |
| Moryella | *Moryella sp.* HOT097 | 0.088 | 0.015 |  | 0.054 | 0.076 |
|  | *Moryella sp.* HOT419 | 0.091 | 0.032 |  | 0.107 | 0.119 |
|  | *Moryella sp.* HOT910 | 0.000 | 0.000 |  | 0.003 | 0.000 |
| Oribacterium | *Oribacterium sp.* HOT108 | 0.000 | 0.000 |  | 0.003 | 0.024 |
| Parvimonas | *Parvimonas micra* | 0.000 | 0.000 |  | 0.031 | 0.010 |
|  | *Parvimonas sp.* HOT110, HOT393 | 0.023 | 0.024 |  | 0.035 | 0.011 |
|  | *Parvimonas sp.* HOT393 | 0.109 | 0.110 |  | 0.018 | 0.057 |
| Peptococcus | *Peptococcus sp.* HOT167 | 0.010 | 0.004 |  | 0.007 | 0.045 |
|  | *Peptococcus sp.* HOT168. HOT167 | 0.000 | 0.000 |  | 0.023 | 0.037 |
| Peptostreptococcaceae[11][G-2] | *Peptostreptococcaceae [11][G-2] sp.* HOT091 | 0.000 | 0.000 |  | 0.000 | 0.003 |
| Peptostreptococcaceae[11][G-4] | *Peptostreptococcaceae [11][G-4] sp.* HOT369 | 0.000 | 0.000 |  | 0.000 | 0.009 |
| Peptostreptococcaceae[11][G-5] | *Peptostreptococcaceae [11][G-5] sp.* HOT493 | 0.000 | 0.000 |  | 0.000 | 0.007 |
| Peptostreptococcaceae[11][G-7] | *Peptostreptococcaceae [11][G-7] sp.* HOT081 | 0.000 | 0.000 |  | 0.000 | 0.006 |
| Peptostreptococcus | *Peptostreptococcus stomatis* | 0.024 | 0.019 |  | 0.050 | 0.067 |
| Selenomonas | *Selenomonas artemidis* | 0.606 | 0.668 |  | 0.599 | 0.430 |
|  | *Selenomonas dianae* | 0.000 | 0.000 |  | 0.000 | 0.029 |
|  | *Selenomonas flueggei* | 0.017 | 0.004 |  | 0.034 | 0.104 |
|  | *Selenomonas infelix. S. sp.* HOT481 | 0.286 | 0.316 |  | 0.312 | 0.508 |
|  | *Selenomonas noxia* | 0.209 | 0.501 |  | 0.585 | 1.132 |
|  | *Selenomonas sp.* HOT126 | 0.112 | 0.082 |  | 0.110 | 0.377 |
|  | *Selenomonas sp.* HOT133 | 0.000 | 0.000 |  | 0.000 | 0.009 |
|  | *Selenomonas sp.* HOT134 | 0.000 | 0.016 |  | 0.004 | 0.000 |
|  | *Selenomonas sp.* HOT137 | 0.230 | 0.243 |  | 1.163 | 0.302 |
|  | *Selenomonas sp.* HOT140, *S. noxia* | 0.191 | 0.478 |  | 0.830 | 0.218 |
|  | *Selenomonas sp.* HOT146 | 0.107 | 0.077 |  | 0.142 | 0.097 |
|  | *Selenomonas sp.* HOT149 | 0.461 | 0.037 |  | 0.010 | 0.007 |
|  | *Selenomonas sp.* HOT478 | 0.010 | 0.000 |  | 0.001 | 0.000 |
|  | *Selenomonas sp.* HOT892 | 0.215 | 0.429 |  | 0.214 | 0.296 |
|  | *Selenomonas sputigena* | 0.498 | 0.190 |  | 0.105 | 0.147 |
| Shuttleworthia | *Shuttleworthia satelles* | 0.002 | 0.000 |  | 0.007 | 0.000 |
| Streptococcus | *Streptococcus anginosus* | 0.113 | 0.015 |  | 0.624 | 0.080 |
|  | *Streptococcus australis* | 0.102 | 0.030 |  | 0.058 | 0.008 |
|  | *Streptococcus constellatus* | 0.099 | 0.005 |  | 0.080 | 0.047 |
|  | *Streptococcus gordonii* | 5.267 | 6.264 |  | 2.129 | 2.908 |
|  | *Streptococcus intermedius* | 0.224 | 0.090 |  | 0.698 | 0.497 |
|  | *Streptococcus mutans* | 0.019 | 0.056 |  | 0.151 | 0.019 |
|  | *Streptococcus oligofermentans. S.gordonii, S.cristatus, S.australias* | 3.759 | 4.994 |  | 4.423 | 2.997 |
|  | *Streptococcus oralis, S. mitis, S. mitis bv2, S. infantis* | 26.360 | 35.915 |  | 27.157 | 25.903 |
|  | *Streptococcus parasanguinis I S. sp.* HOT066 | 0.006 | 0.013 |  | 0.018 | 0.028 |
|  | *Streptococcus parasanguinis II, S. sp.* HOT065, HOT067 | 0.015 | 0.014 |  | 0.063 | 0.038 |
|  | *Streptococcus peroris. S.sp. HOT068* | 1.558 | 1.581 |  | 2.323 | 2.694 |
|  | *Streptococcus pneumonia, S. oralis, S. mitis, S. infantis* | 0.011 | 0.028 |  | 0.010 | 0.008 |
|  | *Streptococcus salivarius, S. vestibularis* | 0.000 | 0.018 |  | 0.048 | 0.222 |
|  | *Streptococcus sanguinis* | 10.011 | 5.873 |  | 7.753 | 6.334 |
|  | *Streptococcus sinensis, S. peroris, S. parasanguinis II* | 0.015 | 0.023 |  | 0.031 | 0.032 |
|  | *Streptococcus sp.* HOT056 | 0.337 | 0.630 |  | 0.181 | 0.388 |
|  | *Streptococcus sp.* HOT057 | 0.000 | 0.000 |  | 0.001 | 0.003 |
|  | *Streptococcus sp.* HOT068, *S. peroris* | 0.000 | 0.010 |  | 0.028 | 0.008 |
|  | *Streptococcus sp.* HOT071 | 0.007 | 0.014 |  | 0.010 | 0.011 |
|  | *Streptococcus sp.* HOT074 | 0.163 | 0.117 |  | 0.084 | 0.103 |
|  | *Streptococcus sp.* HOT431 | 0.002 | 0.017 |  | 0.004 | 0.003 |
| Veillonella | *Veillonella atypica* | 0.005 | 0.007 |  | 0.016 | 0.008 |
|  | *Veillonella dispar* | 3.972 | 2.115 |  | 3.010 | 4.288 |
|  | *Veillonella parvula* | 0.738 | 0.719 |  | 1.384 | 4.072 |
|  | *Veillonella rogosae* | 0.038 | 0.061 |  | 0.093 | 0.085 |
|  | *Veillonella sp. HOT780* | 0.324 | 0.795 |  | 0.148 | 0.404 |
| Veillonellaceae[G-1] | *Veillonellaceae [G-1] sp.* HOT129 | 0.000 | 0.000 |  | 0.000 | 0.004 |
|  | *Veillonellaceae [G-1] sp.* HOT132, | 0.045 | 0.017 |  | 0.004 | 0.001 |
|  | *Veillonellaceae [G-1] sp.* HOT155 | 0.005 | 0.003 |  | 0.009 | 0.004 |
| Fusobacterium | *Fusobacterium naviforme* | 0.083 | 0.015 |  | 0.137 | 0.167 |
|  | *Fusobacterium nucleatum ss polymorphum* | 2.332 | 2.257 |  | 3.719 | 2.919 |
|  | *Fusobacterium nucleatum ss vincentii* | 0.215 | 0.037 |  | 0.640 | 0.265 |
|  | *Fusobacterium nucleatum ss. animalis* | 0.249 | 0.114 |  | 0.735 | 0.160 |
|  | *Fusobacterium nucleatum ss. Nucleatum, F. sp.* HOT203 | 0.016 | 0.022 |  | 0.047 | 0.069 |
|  | *Fusobacterium periodonticum* | 0.968 | 0.220 |  | 0.564 | 0.431 |
|  | *Fusobacterium sp.* HOT203 | 0.060 | 0.019 |  | 0.491 | 0.329 |
|  | *Fusobacterium sp.* HOT203, HOT370 | 0.025 | 0.005 |  | 0.171 | 0.093 |
| Leptotrichia | *Leptotrichia buccalis* | 0.002 | 0.185 |  | 0.457 | 0.189 |
|  | *Leptotrichia goodfellowii* | 0.035 | 0.014 |  | 0.101 | 0.046 |
|  | *Leptotrichia hofstadii* | 0.179 | 0.232 |  | 1.023 | 0.295 |
|  | *Leptotrichia hongkongensis* | 0.197 | 0.296 |  | 0.579 | 0.801 |
|  | *Leptotrichia shahii* | 0.000 | 0.007 |  | 0.097 | 0.034 |
|  | *Leptotrichia sp.* HOT212 | 1.113 | 0.759 |  | 1.174 | 1.431 |
|  | *Leptotrichia sp.* HOT215 | 0.167 | 0.118 |  | 0.311 | 0.281 |
|  | *Leptotrichia sp.* HOT219 | 0.003 | 0.031 |  | 0.336 | 0.187 |
|  | *Leptotrichia sp.* HOT221 | 0.009 | 0.003 |  | 0.162 | 1.036 |
|  | *Leptotrichia sp.* HOT223 | 0.024 | 0.024 |  | 0.066 | 0.062 |
|  | *Leptotrichia sp.* HOT225, *L. buccalis* | 0.115 | 1.553 |  | 0.530 | 0.201 |
|  | *Leptotrichia sp.* HOT392 | 0.132 | 0.097 |  | 0.126 | 0.152 |
|  | *Leptotrichia sp.* HOT417, HOT462 | 0.006 | 0.051 |  | 0.080 | 0.227 |
|  | *Leptotrichia sp.* HOT463 | 0.002 | 0.022 |  | 0.031 | 0.093 |
|  | *Leptotrichia sp.* HOT498 | 0.002 | 0.000 |  | 0.000 | 0.002 |
|  | *Leptotrichia sp.* HOT879 | 0.005 | 0.002 |  | 0.004 | 0.148 |
|  | *Leptotrichia wadei* | 0.000 | 0.009 |  | 0.163 | 0.210 |
| Aggregatibacter | *Aggregatibacter actinomycetemcomitans* | 0.000 | 0.000 |  | 0.003 | 0.000 |
|  | *Aggregatibacter aphrophilus* | 0.004 | 0.018 |  | 0.012 | 0.095 |
|  | *Aggregatibacter paraphrophilus* | 0.296 | 2.126 |  | 0.984 | 1.670 |
|  | *Aggregatibacter segnis* | 0.035 | 0.576 |  | 0.199 | 0.058 |
|  | *Aggregatibacter sp.* HOT458 | 0.138 | 0.076 |  | 0.161 | 0.276 |
|  | *Aggregatibacter sp.* HOT513 | 0.032 | 0.086 |  | 0.050 | 0.403 |
|  | *Aggregatibacter sp.* HOT898 | 0.000 | 0.035 |  | 0.015 | 0.074 |
| Campylobacter | *Campylobacter concisus* | 0.025 | 0.088 |  | 0.334 | 0.161 |
|  | *Campylobacter curvus* | 0.000 | 0.000 |  | 0.004 | 0.000 |
|  | *Campylobacter gracilis* | 0.093 | 0.088 |  | 0.349 | 0.303 |
|  | *Campylobacter rectus* | 0.168 | 0.251 |  | 0.187 | 0.204 |
| Cardiobacterium | *Cardiobacterium hominis* | 0.007 | 0.068 |  | 0.036 | 0.078 |
|  | *Cardiobacterium valvulum* | 0.009 | 0.011 |  | 0.015 | 0.009 |
| Eikenella | *Eikenella corrodens* | 0.021 | 0.022 |  | 0.027 | 0.062 |
| Haemophilus | *Haemophilus haemolyticus* | 0.010 | 0.035 |  | 0.017 | 0.020 |
|  | *Haemophilus influenzae* | 0.010 | 0.033 |  | 0.082 | 0.062 |
|  | *Haemophilus parainfluenzae* | 3.316 | 2.351 |  | 5.960 | 2.559 |
|  | *Haemophilus sp.* HOT035 | 0.152 | 0.067 |  | 0.025 | 0.026 |
|  | *Haemophilus sp.* HOT036 | 0.012 | 0.022 |  | 0.008 | 0.109 |
|  | *Haemophilus sp.* HOT908 | 0.000 | 0.012 |  | 0.003 | 0.004 |
| Kingella | *Kingella denitrificans* | 0.036 | 0.015 |  | 0.022 | 0.109 |
|  | *Kingella oralis* | 0.082 | 0.057 |  | 0.172 | 0.337 |
|  | *Kingella sp.* HOT012 | 0.000 | 0.006 |  | 0.000 | 0.002 |
| Lautropia | *Lautropia mirabilis* | 0.124 | 0.227 |  | 0.289 | 0.209 |
| Neisseria | *Neisseria elongata* | 0.057 | 0.345 |  | 0.123 | 0.643 |
|  | *Neisseria flavescens* | 0.126 | 0.062 |  | 0.079 | 0.220 |
|  | *Neisseria mucosa* | 1.156 | 0.330 |  | 0.105 | 0.428 |
|  | *Neisseria pharyngis* | 0.044 | 0.506 |  | 0.288 | 0.743 |
|  | *Neisseria sp.* HOT014, HOT016 | 0.005 | 0.160 |  | 0.219 | 0.042 |
|  | *Neisseria subflava* | 0.002 | 0.026 |  | 0.025 | 0.164 |
| Ottowia | *Ottowia sp.* HOT894 | 0.000 | 0.005 |  | 0.034 | 0.084 |
| SR1[G-1] | *SR1 [G-1] sp.* HOT345 | 0.000 | 0.000 |  | 0.000 | 0.010 |
|  | *SR1 [G-1] sp.* HOT874 | 0.030 | 0.141 |  | 0.163 | 0.215 |
|  | *SR1 [G-1] sp.* HOT875 | 0.049 | 0.031 |  | 0.062 | 0.108 |
| Fretibacterium | *Fretibacterium fastidiosum* | 0.011 | 0.044 |  | 0.008 | 0.020 |
|  | *Fretibacterium sp.* HOT360 | 0.000 | 0.000 |  | 0.000 | 0.004 |
| Mycoplasma | *Mycoplasma faucium* | 0.000 | 0.000 |  | 0.000 | 0.007 |
| TM7[G-1] | *TM7 [G-1] sp.* HOT346 | 0.002 | 0.000 |  | 0.009 | 0.006 |
|  | *TM7 [G-1] sp.* HOT347 | 0.000 | 0.004 |  | 0.000 | 0.012 |
|  | *TM7 [G-1] sp.* HOT348 | 0.000 | 0.000 |  | 0.003 | 0.008 |
|  | *TM7 [G-1] sp.* HOT349 | 0.000 | 0.000 |  | 0.002 | 0.000 |
|  | *TM7 [G-1] sp.* HOT353 | 0.010 | 0.004 |  | 0.011 | 0.000 |
| TM7[G-2] | *TM7 [G-2] sp.* HOT350 | 0.000 | 0.000 |  | 0.005 | 0.000 |
| TM7[G-3] | *TM7 [G-3] sp.* HOT351 | 0.005 | 0.007 |  | 0.039 | 0.002 |
| TM7[G-4] | *TM7 [G-4] sp.* HOT355 | 0.023 | 0.013 |  | 0.008 | 0.012 |
